# Neurally mediated syncope diagnosis based on adenylate cyclase activity in Japanese patients

**DOI:** 10.1371/journal.pone.0214733

**Published:** 2019-04-18

**Authors:** Tomoyoshi Komiyama, Eiichiro Nagata, Tadashi Hashida, Susumu Sakama, Kengo Ayabe, Hiroshi Kamiguchi, Ayumi Sasaki, Koichiro Yoshioka, Hiroyuki Kobayashi

**Affiliations:** 1 Department of Clinical Pharmacology, Tokai University School of Medicine, 143 Shimokasuya, Isehara, Kanagawa, Japan; 2 Department of Neurology, Tokai University School of Medicine, 143 Shimokasuya, Isehara, Kanagawa, Japan; 3 Department of Cardiovascular Internal Medicine, Tokai University School of Medicine, 143 Shimokasuya, Isehara, Kanagawa, Japan; 4 Support Center for Medical Research and Education, Tokai University, 143 Shimokasuya, Isehara, Kanagawa, Japan; International University of Health and Welfare, School of Medicine, JAPAN

## Abstract

The study aims to clarify the mechanism in patients with neurally mediated syncope (NMS), focusing on the adenylate cyclase (AC) activity level in lymphocytes. This study included 40 subjects: 22 healthy volunteers and 18 NMS patients. We investigated the changes in AC activity that occur during of syncope at rest and during the head-up tilt (HUT) test. We obtained 8 mL of blood at rest time and four times during the HUT test. Then, we measured the AC activity and the test reagent was added to the lymphocytes (10,000) and reacted for 30 min at room temperature. We were able to determine the standard value of AC activity when adrenaline (AD) and isoproterenol (IP) were added to lymphocytes. The results of our study showed one of the causes of NMS has a difference in AC activity level and classification of the patients into two different types of NMS was possible: either the vasodepressor type (VT) or mixed type (MT). At rest time, VT patients showed significantly higher AC activity (AD; 100 μM: *p* = 0.005, IP; 50 μM: *p* = 0.02) and MT patients showed significantly lower AC activity (AD; 10 μM: *p* = 0.02, IP; 50 μM: *p* = 0.004) than the average AC activity in healthy volunteers. Moreover, VT patients had significantly higher AC activity than healthy volunteers at the four points of the HUT test. MT patients had significantly lower AC activity (AD: *p* = 0.04 and IP: *p* = 0.04) than healthy volunteers at the rest time of HUT. Our study showed a significant difference in AC activities between NMS patients and healthy volunteers at rest. Therefore, a detailed NMS diagnosis can be made by examining AC activity levels in blood taken at rest time.

## Introduction

In this study, we sought to clarify the mechanism of neurally mediated syncope (NMS). In recent years, approximately 20% of unknown syncope cases were considered to be NMS [1–3]. Frequently occurring NMS lead to problems in everyday life [4, 5]. The underlying mechanisms causing NMS have yet to be clarified before any treatment can be proposed to patients.

In general, NMS patients are treated lightly as they quickly recover consciousness. However, while NMS itself is not fatal, NMS patients can experience secondary damage (head or chest contusion etc.) as a result of collapse [6–9]. Moreover, when the individual affected by NMS are drivers, this can lead to dangerous situations involving other people [6, 10, 11]. Regarding the impact on the patient and his environment, NMS needs to be considered a serious disease, its causes clarified, and proper diagnosis methods developed. In the NMS diagnosis, understanding the cause of symptoms is very important, such as for instance fainting in a standing or sitting position. However, since many diseases can result in fainting spells (e.g., orthostatic hypotension, cardiogenic syncope, arrhythmia, epilepsy, and cerebral vascular disorders), various additional examinations are required to confirm syncope-related symptoms [12–18]. If NMS is suspected, diagnosis will entail conducting a head-up tilt (HUT) test [19–22]. The HUT test will classify the NMS into one of the three different types: the vasodepressor type (VT), mixed type (MT), and cardioinhibitory type (CT) [23–27].

AC mediates the effects of the Gi protein on the contraction and relaxation of blood vessels by acting on α2B-AR [28–38]. When activated, the β2 receptor promotes the binding of AC to the Gs protein to generate cAMP [39–43]. When cAMP activates protein kinase A, calcium ion channels are opened, accelerating calcium uptake by the sarcoplasmic reticulum [40, 44–46]. Consequently, increased calcium concentrations lead to an increase in the contractile force of smooth muscle [47–50]. Therefore, we speculated that this signaling affects AC activity related to the induction of NMS.

Then, we analyzed the mechanism of the molecular interaction and genetic polymorphisms in the adrenergic receptor (α2B-AR) gene. In particular, we focused on the glutamic acid repeat polymorphism site at Glu 301–303 in the α2B-AR gene, and evaluated the phenotypes of Glu12/12, Glu12/9, and Glu9/9. By measuring the difference in the signaling time of the Gi-α subunit (GPCR), we found that the binding energy of the Gi α-subunit in Glu9 was stronger than that in Glu12 [51]. In addition, Small (2001) et al. reported that both Glu 9 and Glu12 repeats showed different AC activity in ovary cells of the Chinese hamster [52]. Thus, we primarily investigated when changes in AC activity levels markedly occur at the NMS.

Past clinical studies have investigated whether AC activity is associated with NMS in patients. We propose to test AC activity in healthy volunteers and NMS patients. Moreover, we investigate the causes of NMS syncope to develop an early diagnosis method that could be used to diagnose NMS in otherwise healthy people.

## Material and methods

### Ethics statement

The respective institutional ethics committees approved our studies. All the subjects gave written informed consent, including for clinical research and AC activity studies.

———————————————————————————————

**Tokai University**, **School of Medicine IRB**

**Institutional Review Board for Medical Ethics**

**Chairman**;

**Institutional Review Board for Clinical Research**, **Tokai University**

1. Chairman Dr. Munetaka Haida *Tokai University Junior College* of Nursing and *Technology* University president

2. Vice Chairman Dr. Hiroyuki Kobayashi, Department of Clinical Pharmacology, Tokai University School of Medicine, Professor

3. Dr. Rumiko Shimazawa, Department of Clinical Pharmacology, Tokai University School of Medicine, Professor

4. Dr. Kei Takeshita, Department of Medical Ethics, Tokai University School of Medicine, Professor

5. Dr. Naoki Yazawa, Department of gastroenterological surgery, Tokai University School of Medicine, Associate Professor

6. Dr. Masao Toyoda, Division of Nephrology and Metabolism, Department of Internal MedicineTokai University School of Medicine, Associate Professor

7. Dr. Daisuke Sakai, Department of Orthopaedic Surgery, Tokai University School of Medicine, Associate Professor

8. Dr. Kenji Yokoyama, Department of Hematology and Oncology, Tokai University School of Medicine, Associate Professor

9. Dr. Yuji KOYAMA, Department of Rehabilitation Sciences, Tokai University School of Medicine, Associate Professor

10. Dr. Noriaki Kishimoto, Health Management Science, Tokai University School of Medicine, Senior Lecturer

11. Dr. Takashi Tsukamoto, Department of law department, Tokai University

12. Dr. Michio Oshikubo, Department of law department, Tokai University

13. M.S. Meiko Okabe, Department of Nursing, Tokai University School of Medicine, Associate Professor

14. Tsuyoshi Kimura, Medical Division, Deputy director

15. Koichiro Sato, Pharmaceutical department, Pharmacy technician

16. Shuichi Ishida, Academic Research Support Division, Manager

17. Kazumi Nitta, Nursing unit, Tokai University Hospital

18. Dr. Ayako Mikami, Center Hospital of National Center for Global Health and Medicine, Department, Off-Campus Committee

19. Dr. Nobuhiro Sudo, Hatano Isehara Medical Association, Chairman, Off-Campus Committee

20. Kenichi Takahashi, Isehara city hall, Ministry of Health and Welfare, Manager, Off-Campus Committee

21. Reiko Hirai, Isehara city hall, Ministry of Health and Welfare, Chief clerk, Off-Campus Committee

----------------------------------------------------------------

### NMS patients and healthy volunteers

Eighteen Japanese NMS patients (11 males: 42.2 ± 21.2 yr; 7 females: 45.3 ± 21.8 yr) and 22 Japanese healthy volunteers (11 males: 33.5 ± 7.7yr; 11 females: 36.4 ± 7.2 yr) were recruited from the Tokai University School of Medicine from June 2015 to January 2018 (Tables 1, 2, 3 and 4). For the HUT test, 12 healthy volunteers were selected from the 22 healthy volunteers (5 males: 32.4 ± 7.4 yr; 7 females: 38.6 ± 6.3 yr). The diagnoses of NMS patients were based on Colman, et al.[53] and the guidelines of the Japanese Circulation Society (http://www.j-circ.or.jp/english/). The type of NMS (vasodepressor type: VT, mixed type: MT, and cardioinhibitory type: CT) was diagnosed based on data obtained in the electrocardiogram, blood pressure, and pulse at the tilt test. All NMS patients fainted in the tilt test [54]. Organic disease was excluded.

**Table 1:** Characteristics of the 22 healthy volunteers.

	Date	No.	Age	Sex	Systolic BP mmHg (Upper)	Diastolic BP mmHg (Lower)	Pulse
1	2015/6/22	15C015	23	Male	111	67	69
2	2015/8/7	15C019	32	Male	118	67	65
3	2015/9/17	15C021	32	Male	105	56	57
4	2016/4/19	15C032	40	Male	120	79	85
5	2016/9/29	15C041*	43	Male	114	75	63
6	2016/12/1	15C045	48	Male	117	83	82
7	2016/12/16	15C046*	28	Male	117	76	66
8	2017/1/13	15C047*	25	Male	111	59	74
9	2016/8/26	15C040*	29	Male	114	68	52
10	2015/12/11	15C027	31	Male	109	70	83
11	2018/1/19	15C053*	37	Male	116	83	67
12	2015/6/5	15C011	30	Female	99	67	65
13	2015/6/5	15C012	28	Female	96	58	65
14	2015/9/17	15C022*	39	Female	96	64	71
15	2016/2/29	15C031	44	Female	116	79	68
16	2016/6/9	15C034	28	Female	81	55	67
17	2016/6/24	15C036*	47	Female	106	71	64
18	2016/6/30	15C037*	42	Female	108	64	71
19	2016/7/7	15C038*	42	Female	91	48	54
20	2016/12/1	15C044*	37	Female	119	76	72
21	2017/3/10	15C048*	36	Female	109	67	87
22	2017/8/4	15C051*	27	Female	93	60	78

* Implemented the HUT test as the healthy volunteers.

**Table 2:** Average and SD of the 22 healthy volunteers.

	Age	Systolic BP mmHg (Upper)	Diastolic BP mmHg (Lower)	Pulse
Male (Average)	33.45	113.82	71.18	69.36
SD: standard deviation	7.74	4.45	8.96	10.69
Female (Average)	36.36	101.27	64.45	69.27
SD: standard deviation	7.15	11.42	9.07	8.42

**Table 3:** Characteristics of the 18 NMS patients.

	Date	No.	NMS Type	Age	Sex	Systolic BP mmHg (Upper)	Diastolic BP mmHg (Lower)	Pulse
1	2016/3/22	15S006	Cardioinhibitory type	20	Male	102	55	56
2	2016/9/15	15S014	Mixed type	23	Male	119	64	63
3	2017/1/31	15S018	Vasodepressor type	55	Male	108	75	44
4	2017/4/4	15S022	Vasodepressor type	19	Male	127	69	68
5	2017/4/12	15S023	Mixed type	58	Male	130	85	73
6	2017/4/25	15S025	Vasodepressor type	67	Male	129	81	66
7	2017/7/13	15S027	Vasodepressor type	42	Male	115	80	55
8	2017/9/14	15S028	Vasodepressor type	21	Male	114	66	77
9	2017/12/21	15S030	Vasodepressor type	68	Male	127	76	52
10	2018/1/16	15S033	Vasodepressor type	67	Male	90	58	56
11	2018/3/13	15S038	Vasodepressor type	24	Male	108	71	56
12	2016/5/17	15S010	Vasodepressor type	70	Female	116	79	47
13	2016/7/26	15S011	Vasodepressor type	59	Female	111	73	48
14	2016/8/2	15S012	Mixed type	47	Female	110	69	69
15	2016/8/9	15S013	Mixed type	27	Female	94	55	64
16	2017/2/21	15S020	Vasodepressor type	20	Female	142	60	95
17	2018/7/31	15S039	Mixed type	70	Female	113	75	54
18	2018/9/18	15S040	Mixed type	24	Female	99	65	51

**Table 4:** Average and SD of the 18 NMS patients.

	Age	Systolic BP mmHg (Upper)	Diastolic BP mmHg (Lower)	Pulse
Male (Average)	42.18	115.36	70.91	60.55
SD: standard deviation	21.19	12.71	9.60	9.78
Female (Average)	45.29	112.14	68.00	61.14
SD: standard deviation	21.75	15.36	8.54	17.04

### HUT test and blood collection

**Fig 1:**
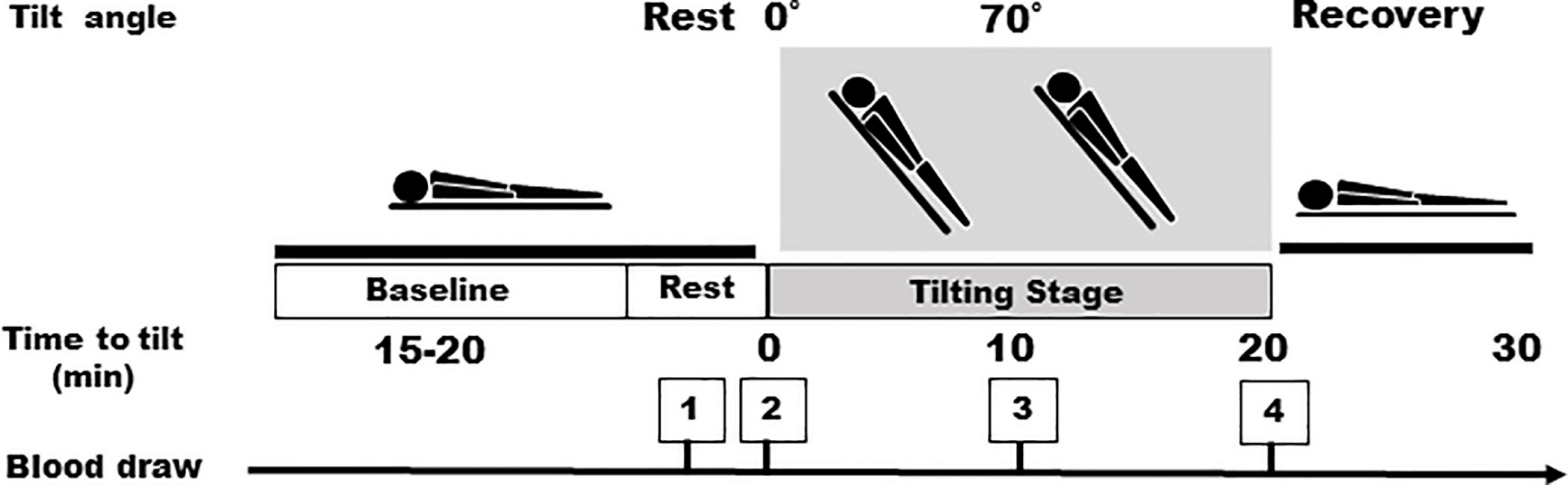
The head-up tilt (HUT) table and points of blood draw.

All the study patients were admitted to the hospital overnight. Patients, not sedated and fasting, underwent the HUT table test between 9:00 am and 11:00 am on a tilt table set to 70°, having rested in the supine position for 15–20 min prior to recording rest time measurements. Healthy volunteers also underwent HUT testing while fasting (Fig 1). The lights and air conditioning were turned off for the duration of the test. Blood was collected from subjects lying on the back for the resting blood draw and we collected 8 mL of blood four times during the HUT test: at the rest time, at 70°, after 10 min, and after 20 min. After removing approximately 1 mL of blood, we switched syringes and drew another 8 mL. To avoid a false positive response, no pharmacological provocation was used and none of the patients had ingested caffeine or medication the day before the test. A positive outcome was defined as the development of syncope or presyncope in association with significant arterial hypotension. We recorded blood pressure, heart rate, and electrocardiogram readings during the HUT testing (Fig 1, Tables 1, 2, 3 and 4).

### Comparative analysis of AC activity with changing timing

We measured changes of the AC activity at rest in three healthy volunteers (C11, C38, C41) during different seasons. Volunteer C38 was tested on August 17^th^, 2015 and on July 7^th^, 2016. Volunteer C11was tested on June 5^th^, and after five months on November 9^th^, 2015. Volunteer C41 was tested on July15^th^, then two months later on September 9^th^, 2016. Moreover, C41 did the HUT test on July 15^th^ and on September 29^th^, 2016.

### Measurement of AC activity and preparation of lymphocytes

**Fig 2:**
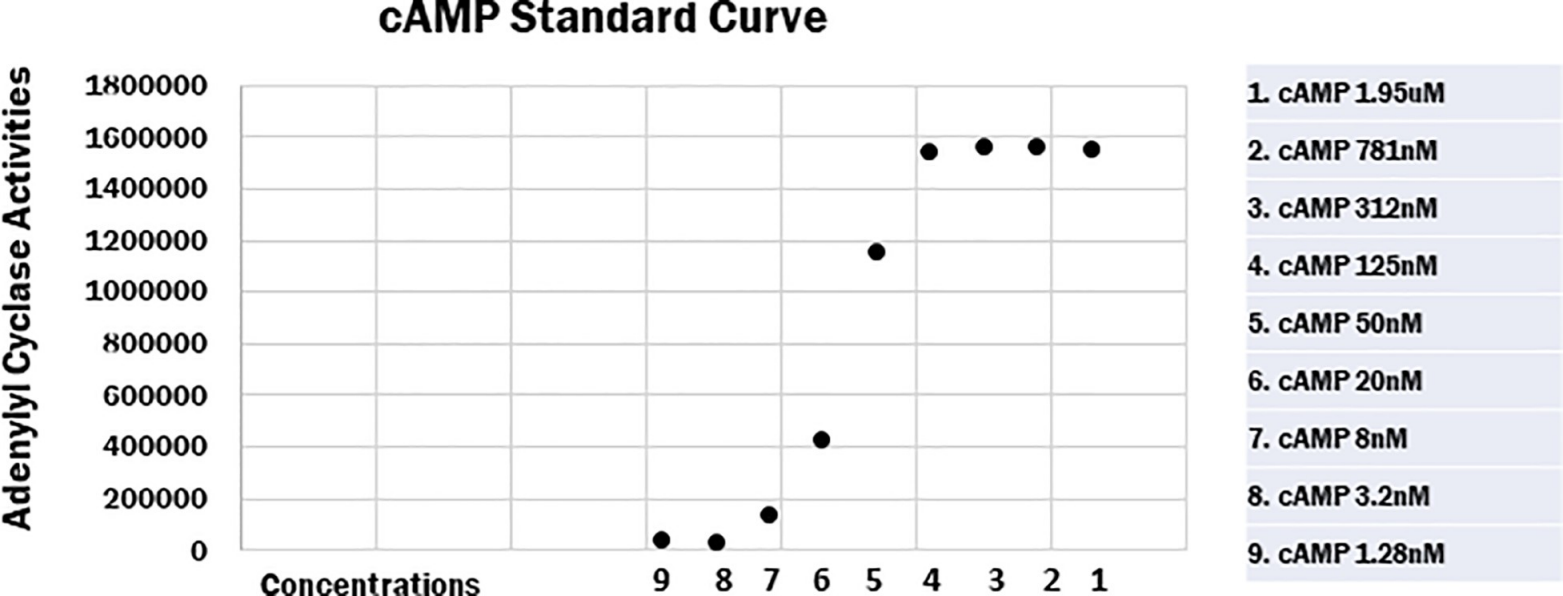
Standard and forskolin curves of cAMP concentrations. A standard curve of the cAMP concentrations.

In order to investigate the AC activity amount, we conducted the following experimental method. We isolated the lymphocyte layer from the blood by centrifuge using BD Vacutainer Blood Collection Tubes (New Jersey, USA). Next, we washed the cells with induction Buffer (RPMI 1640, Thermo Fisher Scientific, Inc., Waltham, MA, USA) in a medium to separate platelets and isolate the lymphocytes. Test reagent was added to the lymphocytes (10 μM for 10,000 cells, respectively) and for a reaction time of 30 min at room temperature. Then, we measured the amount of cyclic adenosine monophosphate (cAMP) in accordance with the Promega cAMP-Glo Assay protocol (GloMax-Multi Detection System, Wisconsin, USA). Amounts of cAMP were confirmed in the standard curve (Fig 2 and S1 Table). AC activities were measured in the presence of induction Buffer and /or test reagents (basal) and 100 uM FK. In our study, a concentration of 100 uM FK was reached as a plateau. The results of isoproterenol (IP) and adrenaline (AD) activity levels are expressed as percentages of forskolin (FK)-stimulated activity [52, 55].

### Adjustment of reagent concentrations for assays (adrenaline, isoproterenol, forskolin)

**Fig 3:**
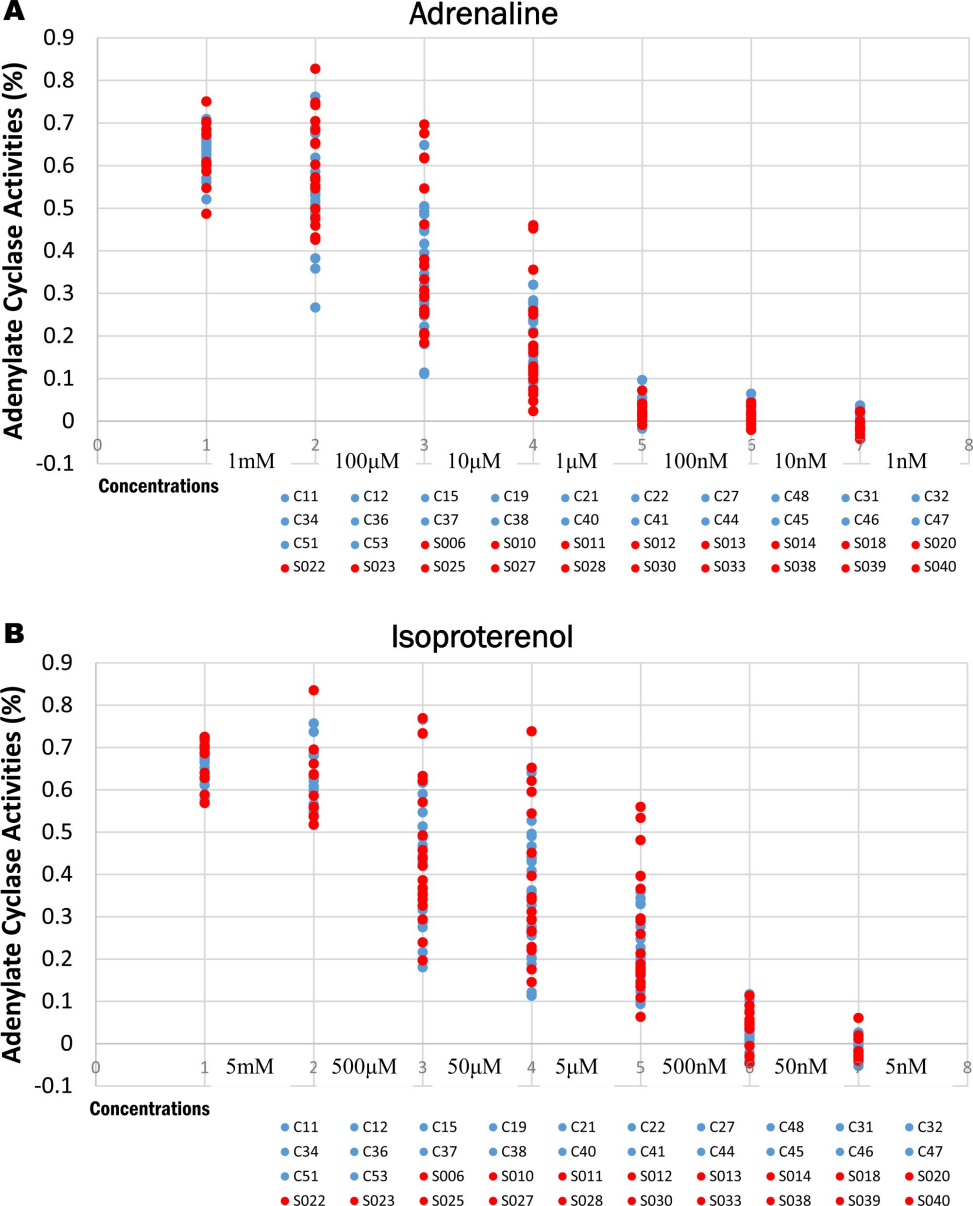
**3A and 3B. Adenylate cyclase activity levels of the 40 subjects.** A: Adrenaline was reduced by 1 mM concentration for each 1/10 at the following concentrations: 1 nM, 10 nM, 100 nM, 1 μM, 10 μM, 100 μM, and 1 mM. The circles suggested 40 total subjects (S2 Table). B: Isoproterenol was decreased by 5 mM for each 1/10 at the following concentrations: 5 nM, 50 nM, 500 nM, 5 μM, 50 μM, 500 μM, and 5 mM. The circles suggested 40 total subjects. Analysis of AC activity in 18 NMS subjects (Red circle) and 22 healthy volunteers (Blue circle) at rest by AD and IP (S2 Table). This analysis excluded one CT NMS patient.

AD is a nonselective agonist of all adrenergic receptors, including the major subtypes α and β [29, 33–35]. IP activates AC for the production of the adrenergic subtype β receptor, which promotes the binding of AC to G proteins to generate cAMP [34, 56–58]. FK activates AC in the production of cAMP [59–62]. Therefore, before measuring AC activity, we verified AD and IP concentrations added to lymphocytes. AD was reduced by 1 mM concentration for each 1/10 at the following concentrations: 1 nM, 10 nM, 100 nM, 1 μM, 10 μM, 100 μM, and 1 mM. IP was decreased by 5 mM for each 1/10 at the following concentrations: 5 nM, 50 nM, 500 nM, 5 μM, 50 μM, 500 μM, and 5 mM. FK was reduced by 100 μM for each 1/5 at the following concentrations: 100 uM, 20 uM, 4 uM, 800 nM, 160 nM, and 32 nM (Fig 3A and 3B).

### Statistical analysis of AC activities

Data are summarized as the mean ± SD. Comparisons were made using *t*-tests as appropriate. Our analysis was performed using Microsoft Office Excel 2019 (ver. 16.10.11029.20045). Statistical analysis was performed using the Excel *t*-test and mean ± standard deviation (SD) program to confirm significant differences. Values of *p* < 0.05 were considered significant. Excel was also used for analyzing the ratio of AC activity amount.

## Results

### Measurement and analysis of AC activity in healthy volunteers and NMS patients at rest

**Fig 4:**
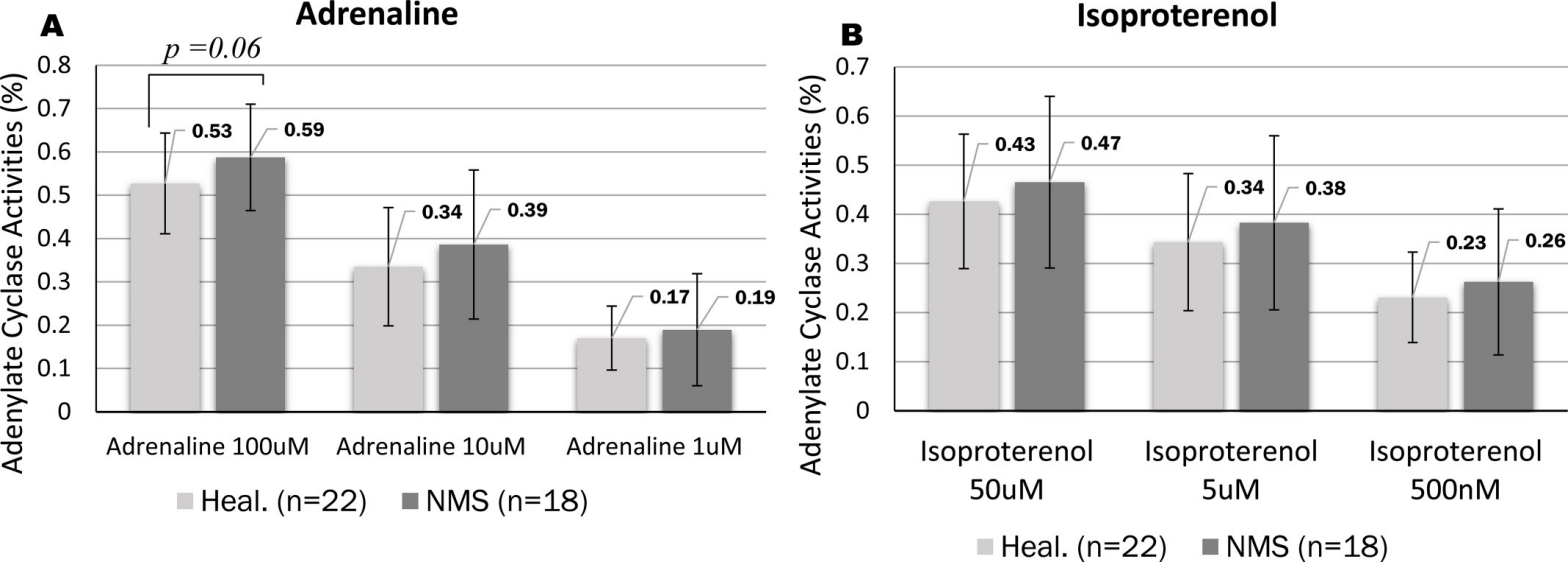
Adenylate cyclase activity in 18 NMS patients and 22 healthy volunteers at rest. A and B are shown for the three concentrations (Adrenaline and Isoproterenol, respectively) of AC activity in healthy volunteers and NMS patients at rest. The AC activity of NMS patients was higher than that of healthy volunteers (AD; 0.54 ± 0.12 for 100 μM, 0.35 ± 0.15 for 10 μM and 0.19 ± 0.10 for 1 μM, IP; 0.44 ± 0.14 for 50 μM, 0.36 ± 0.15 for 5 μM and 0.25 ± 0.11 for 500 nM) at the three concentrations (S2 Table). (A) AD concentrations:100 μM, 10 μM, 1 μM (B) IP concentrations: 50 μM, 5 μM, 500 nM.

We confirmed individual subjects from our results of AC activity with AD and IP (Fig 3A and 3B). Consequently, values between individuals were easily distinguished and observed at the following three concentrations: 1 μM, 10 μM, and 100 μM for AD (Fig 4A) and 500 nM, 5 μM, and 50 μM for IP (Fig 4B). In the 22 healthy volunteers, the average for AD was 0.54 ± 0.12 for 100 μM, 0.35 ± 0.15 for 10 μM, and 0.19 ± 0.10 for 1 μM; the average for IP was 0.44 ± 0.14 for 50 μM, 0.36 ± 0.15 for 5 μM, and 0.25 ± 0.11 for 500 nM. In the 18 NMS patients, the average for AD was 0.59 ± 0.12 for 100 μM, 0.39 ± 0.17 for 10 μM, and 0.19 ± 0.13 for 1 μM; the average for IP was 0.47 ± 0.17 for 50 μM, 0.38 ± 0.18 for 5 μM, and 0.26 ± 0.15 for 500 nM. The AC activity in NMS patients was higher than that in healthy volunteers at the three concentrations tested here. However, AD and IP were not significant in three concentrations between NMS patients and healthy volunteers (AD; 0.54 ± 0.12 for 100 μM, 0.35 ± 0.15 for 10 μM and 0.19 ± 0.10 for 1 μM, IP; 0.44 ± 0.14 for 50 μM, 0.36 ± 0.15 for 5 μM and 0.25 ± 0.11 for 500 nM) (S2 Table). We were then able to determine the standard value of AC activity in the healthy volunteers and the NMS subjects.

### Adenylate cyclase activity in NMS patients at rest

**Fig 5:**
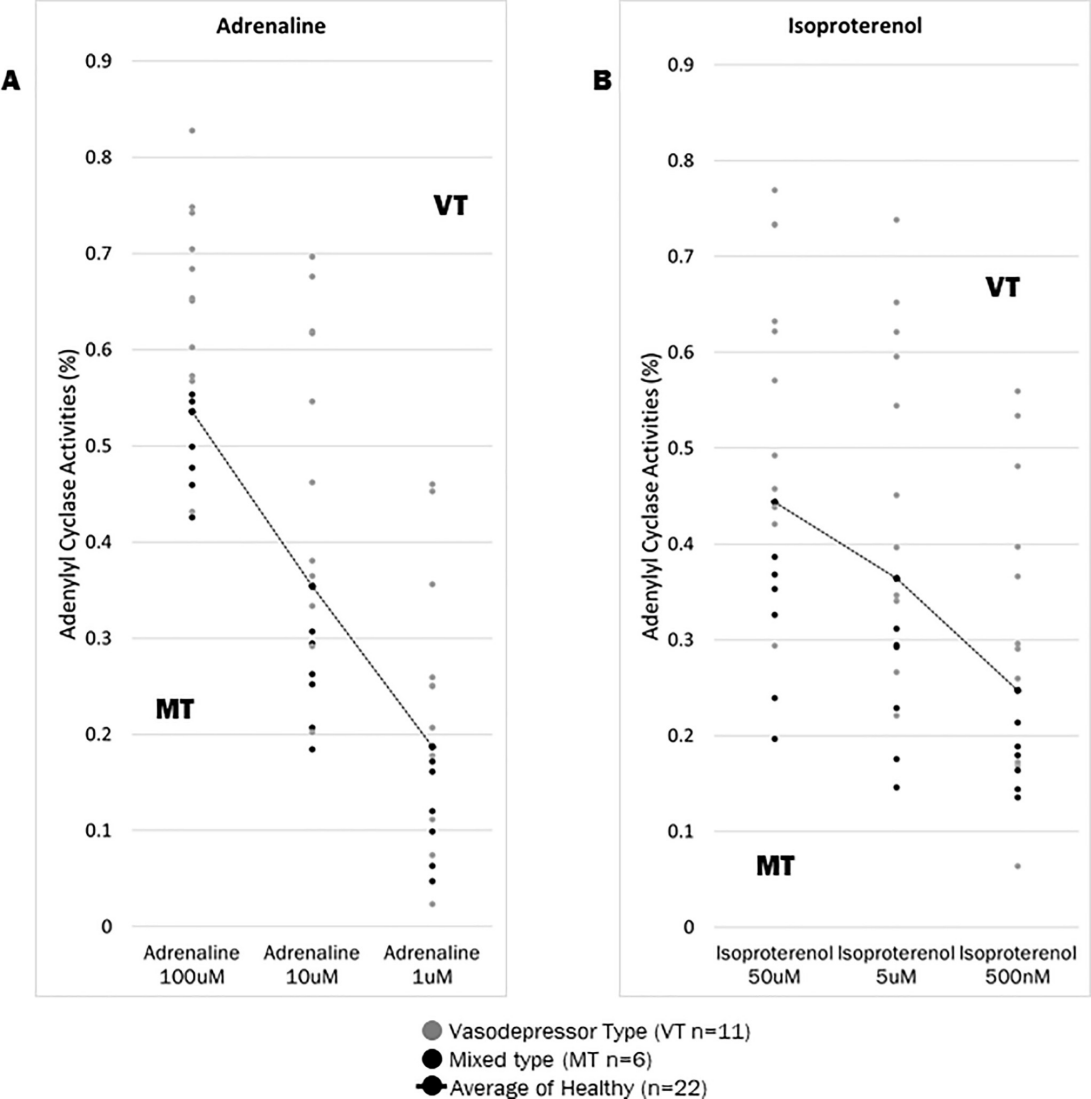
Adenylate cyclase activity in 17 NMS patients at rest. A and B represent the AC activities in 11 vasodepressor type (VT: gray points) and in 6 mixed type (MT: black points) at rest. The data indicated two groups (VT and MT) as border line on average of healthy volunteers (dotted black line) in NMS patients. In particular, this was confirmed at the 10 μM of AD and 5 μM of the IP. The two concentrations have a large variance of standard deviation. Therefore, we analyzed and focused on the three concentrations from the AD and IP data. Error bars show mean ± standard deviation (S2 Table). CT is excluded from Fig 4C and 4D because it has one patient. (A) AD concentrations:100 μM, 10 μM, 1 μM (B) IP concentrations: 50 μM, 5 μM, 500 nM.

**Fig 6:**
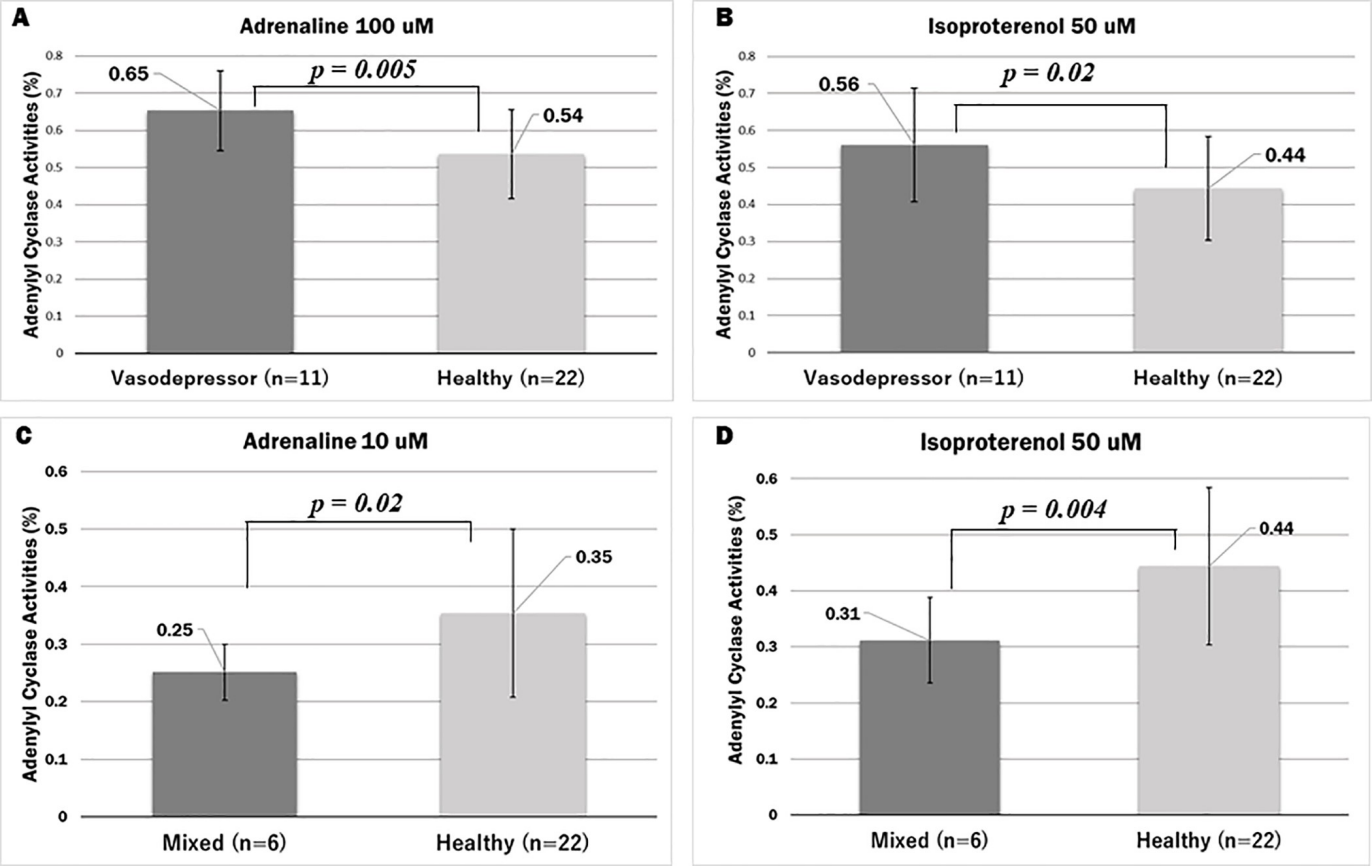
Analysis of AC activities in NMS subjects (VT, MT) and 22 healthy volunteers at rest. This figure shows the *t*-test (*p* < 0.05) among the VT (n = 11), MT (n = 6) of NMS patients and healthy volunteers (n = 22). AD at concentrations 100 μM and 10 μM, IP at 50 μM. Error bars show mean ± standard deviation (S3 Table and S4 Table). (A) AD concentration 100 μM (B and D) IP concentration 50 μM (C) AD concentration 10 μM.

NMS patients were divided into two groups at rest: VT and MT group (Fig 5A and 5B). The VT group had higher AC activity level, while the MT group had lower AC activity levels, compared to the average AC activity level in the healthy volunteer group (S2 Table). The higher group (A) were suggested as the VT of NMS, and the lower group (B) was mainly the MT of NMS. Next, we performed t-tests among the VT group (n = 11) of NMS patients and healthy volunteers (n = 22). From our results, the VT group had significantly higher AC activity with AD; 0.65 ± 0.11 for 100 μM: *p* = 0.005 and 0.47 ± 0.12 for 10 μM: *p* = 0.03 (S3 Table). IP showed significantly higher AC activity in the VT group than in the healthy volunteers at *p* = 0.02 with 0.56 ± 0.15 for 50 μM concentrations (Fig 6A and 6B). When the MT group (n = 6) was compared to healthy volunteers, AC activity was significantly higher in the healthy volunteers than in the NMS patients at AD concentrations of 0.25 ± 0.05 for 10 μM (p = 0.02) and AD concentrations of 0.11 ± 0.05 for 1 μM (p = 0.04). At the three IP concentrations at a 0.31 ± 0.08 for 50 μM (*p* = 0.004), 0.24 ± 0.07 for 5 μM (*p* = 0.005) and 0.17 ± 0.03 for 500 nM (*p* = 0.004) showed significantly lower AC activity in the MT group than in the healthy volunteers (Fig 6C and 6D and S4 Table). At rest time, VT tended to be higher than the average of healthy volunteers, and MT tended to be lower than the average of healthy volunteers.

### Measurement and analysis of AC activity between NMS patients and healthy volunteers during the HUT test

**Fig 7:**
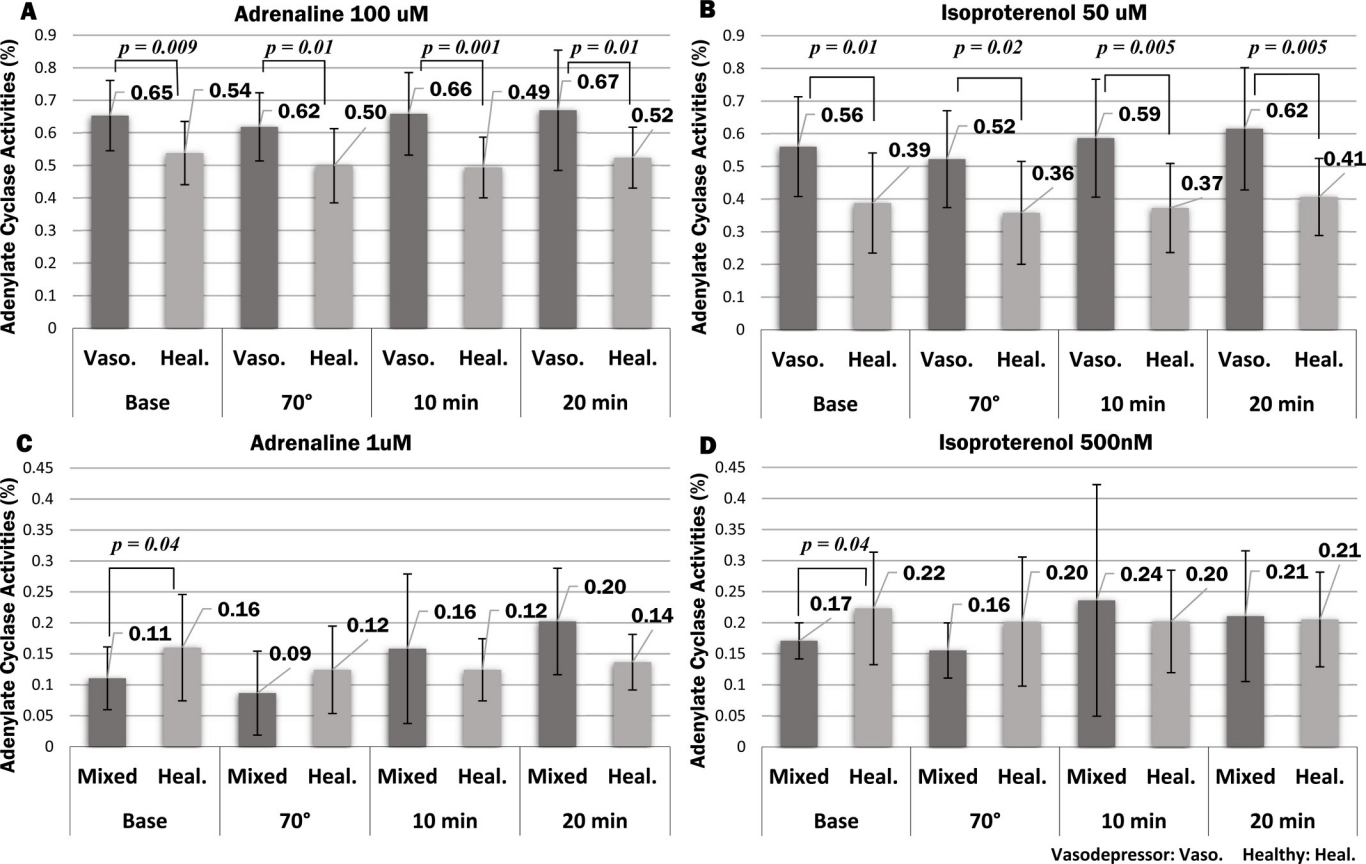
Analysis of AC activities in NMS subjects (VT, MT) and 22 healthy volunteers during the HUT test. This figure shows the analyzed *t*-test (*p* < 0.05) among the VT (n = 11), MT (n = 6) of NMS patients and healthy volunteers (n = 12). AD at concentrations 100 μM and 1 μM, IP at 50 μM. Error bars show mean ± standard deviation (S5 Table and S6 Table). * Some patients were in poor condition around 10 minutes from the tilt table set to 70°at the HUT test. (A) AD concentrations (100 μM) (B) IP concentrations (50 μM) (C) AD concentrations (1μM) (D) IP concentrations (500 nM).

The HUT test was analyzed according to the two different types (VT and MT) of NMS. The four points of the measurement during the HUT test showed that in the VT group (n = 11), the activity of AC with AD and IP was significantly higher than in the healthy volunteers (n = 12). The following results were observed: with 100 μM of AD, base: p = 0.009, at 70°: p = 0.01, after 10 minutes: p = 0.001, and after 20 minutes: p = 0.01; with 50 μM of IP, base: p = 0.01, at 70°: p = 0.02, after 10 minutes: p = 0.005, and after 20 minutes: p = 0.005. Significant differences were found at the other two concentrations. In the MT group, although the AC activity with AD (1 μM: p = 0.04) and IP (500 nM: p = 0.04) was slightly lower in healthy volunteers during the HUT test (rest time), there was either no change or a decrease in AC activity (Fig 7A, 7B, 7C and 7D). However, 10 minutes after the tilt table set to 70°, AC activities had risen sharply, although this was not significant. In the VT and MT groups, AC activity markedly increased 10 minutes after head-up tilt 70° stress, which is thought to be due to presyncope or syncope.

### AC activities in the different seasons from three volunteers at rest and during the HUT test

**Fig 8:**
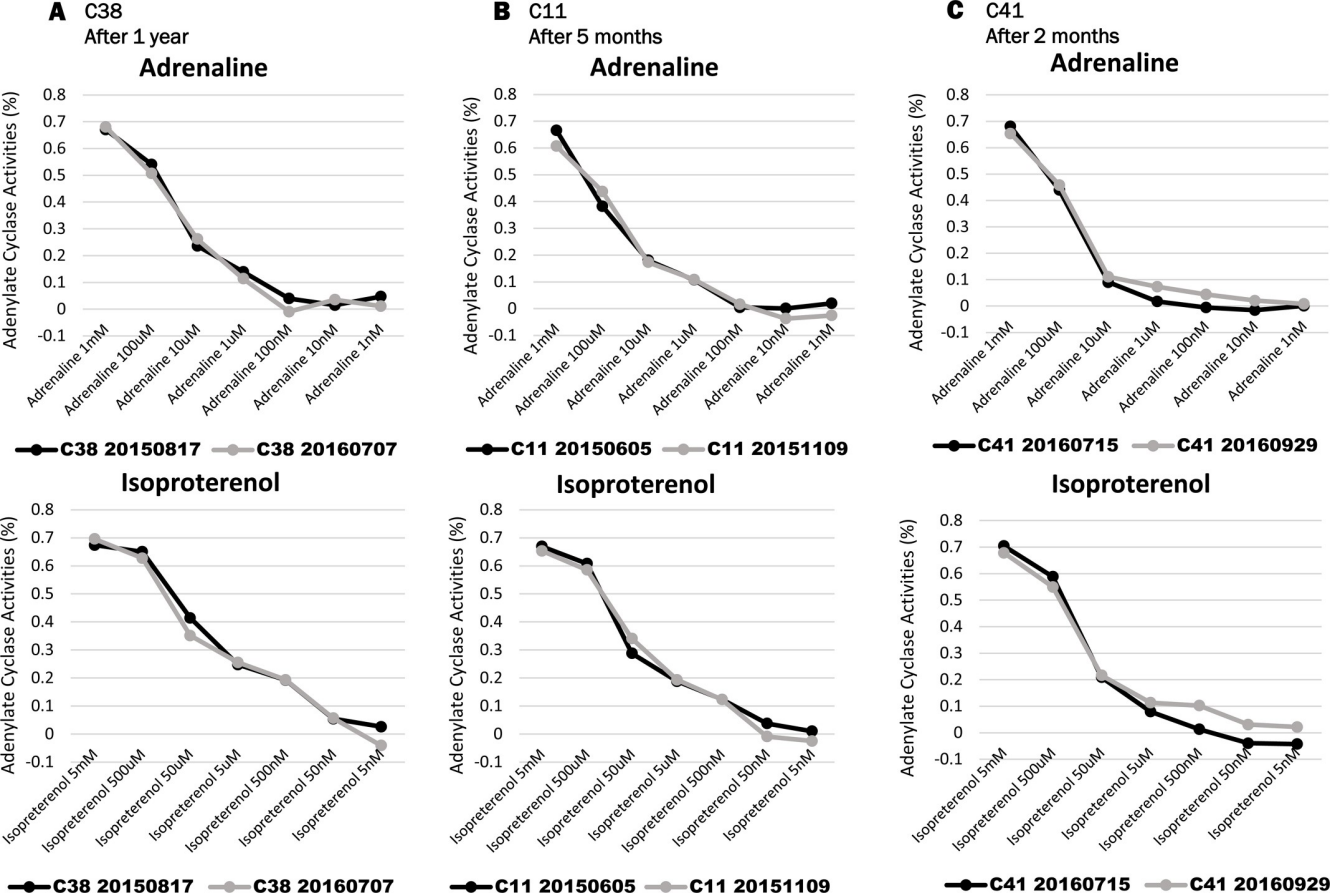
The AC activities during the different seasons from three volunteers at rest. (A) The AC activity in the C38 volunteer was measured on Aug. 17^th^, 2015 and Jul. 7^th^, 2016. (B) The AC activity in the C11 volunteer was measured on Jun. 5^th^, 2015 and Nov. 9^th^, 2015. (C) The AC activity in the C41 volunteer was measured on Jul. 15^th^, 2016 and Sep. 29^th^, 2016. Upper: Adrenaline Lower: Isoproterenol (S7 Table).

**Fig 9:**
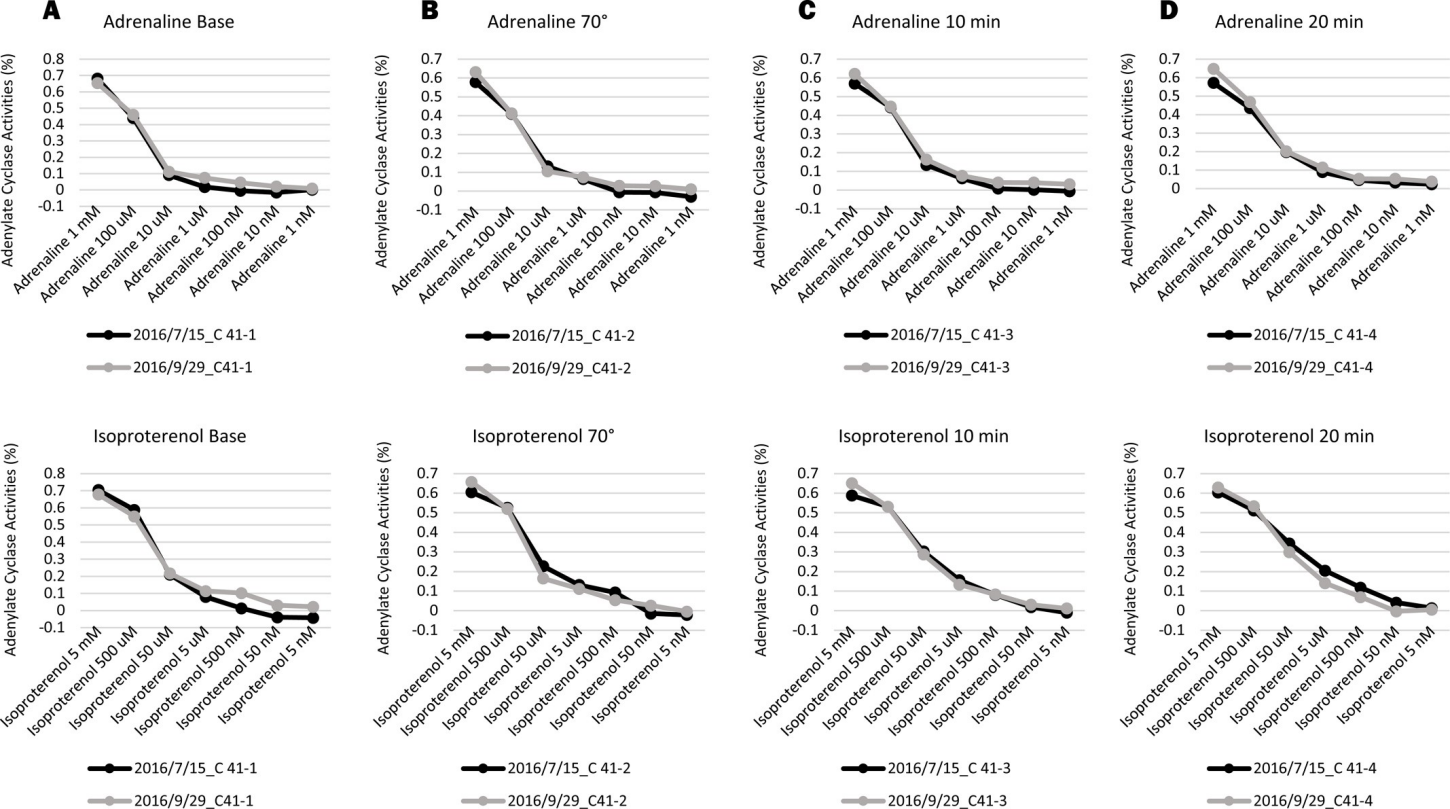
**The AC activities in two different seasons from the C41 volunteer during the HUT test (A: Base, B: 70°, C: 10 min, D: 20 min) (July. 15**^**th**^**, 2016 and Sep. 29**^**th**^**, 2016).** Upper: Adrenaline Lower: Isoproterenol (S8 Table).

In the three healthy volunteers (C11, C38, C41), there was no change in AC activity between different time points (separated by 11 months, five months, or two months) (Fig 8A, 8B and 8C). Moreover, the C41 healthy volunteer did not show a large change during the HUT tests performed at different times. From our results, we do not know exactly if the AC activity values have been decided to each person. However, irrespective of the season, AC activity was similar in each of the three volunteers and the change was minimal in C41 for the two HUT tests (Fig 9A, 9B, 9C and 9D).

## Discussion

To clarify the mechanism of NMS, we focused on the AC activity level at the time of NMS. From our results, we were able to determine the standard value of AC activity when AD and IP were added to lymphocytes. Our study showed a significant difference in AC activity between NMS patients and healthy volunteers at rest and during the HUT test. Interestingly, the NMS types (VT or MT) could be distinguished based on AC activity level by how high or low the activities is. The AC activity (with AD 100 μM and IP 50 μM) is significantly higher in the VT patients than in the healthy volunteers at rest. In addition, the VT was significantly higher than healthy volunteers at the four points of the HUT test. AC activity (AD 10 μM and IP 50 μM) is significantly lower in the MT patients than in the healthy volunteers at rest. We think that the action of the Gi-α (inhibit) or Gs-α (stimulate) subunits are relative to the AC activities occur at the onset of NMS patients. The stimulatory Gs protein activates AC to increase intracellular cAMP. Gi protein inhibit the activation of AC. Therefore, our result suggests that AC activity of VT patients is higher because it is influence by Gi-α subunit and Gs-α subunit. VT patients seems to have relatively stronger action of the Gs-α subunit. In MT patients, the action of Gs-α subunit is weaker, and it is also considered that the AC activity level is lower do to the action of Gi-α subunit. In healthy volunteers, the balance of action between Gi-α and Gs-α subunits has been maintained.

Also, both VT and MT groups, AC activity markedly increased 10 minutes after head-up tilt 70° stress, which is thought to be due to presyncope or syncope. Therefore, we found that it is possible for this data to be used as a diagnosis of NMS patients by AC activity levels. From our results, it became clear that AC activity is strongly related to the appearance of NMS. NMS diagnosis can be determined by examining AC activity levels from blood tests at rest time, without the requirement of performing the HUT test.

In the three healthy volunteers, there was no change in AC activity between HUT tests or between different time points (separated by 11 months, five months, or two months). If the AC activity level can be clarified, and which points at the growth process are determined, it is possible to diagnose a very early risk of NMS patients in the future by conducting AC activity tests at the earliest possible time. Even in environments where it is difficult to diagnose VT and MT, AC activity can become a useful diagnosis tool of NMS.

Based on the different AC activity levels in this study, regarding coping with NMS, it may be necessary to adjust caffeine intake in addition to tilt stretching. Caffeine acts to increase cAMP (AC activity) concentration in cells [63–67]. From our results, patients who have higher AC activity levels than healthy volunteers are at a higher risk of NMS if they ingest caffeine. Conversely, patients with low AC activity may benefit from ingesting food containing caffeine. Because there is no treatment for NMS, suppressing the onset of NMS by adjusting the intake of tea or coffee seems a very easy preventive method.

The final goal of our research is to identify an early diagnosis of NMS and potentially prevent fainting and their associated injuries. Studies examining AC activity in a larger sample size are necessary to confirm the reliability of our findings. Furthermore, in our next report we are discussing the cause of NMS from the multilateral relationship of the glutamic acid repeat polymorphism site at Glu 301–303 in the α2B-AR gene, and evaluated the phenotypes or gene frequency of Glu12/12, Glu12/9, and Glu9/9.

We believe that AC activity tests could be implemented in job applicants for positions involving low gravity stress while operating ultra-high-speed aircraft, linear motor cars, space stations, as well as in long distance truck, bus, and train drivers or an aircraft pilot. In addition, the research in our study may be useful in creating an improved standard of medical management for stress during times of unexpected, large scale disasters in Japan’s future. We believe in the possibility of developing therapeutic drugs for NMS diseases.

## Conclusions

Our study showed a significant difference in AC activities between NMS patients and healthy volunteers at rest and during the HUT test. In addition, we found different patterns among the two NMS types (VT or MT) based on AC activity level. Especially, by examining AC activity level from the blood at rest, it is possible to diagnose NMS patients. Based on our data, we might be able to find people with a high risk of developing NMS from healthy people.

## Supporting information

S1 Table An example raw data of healthy volunteer (C48). (PDF)

S2 Table The raw data of adenylate cyclase activities from 22 healthy volunteers and 18 NMS patients at rest time by adrenaline (AD) and isoproterenol (IP). (PDF)

S3 Table The raw data of adenylate cyclase activities from 22 healthy volunteers and 11 VT patients at rest time by adrenaline (AD) and isoproterenol (IP). (PDF)

S4 Table The raw data of adenylate cyclase activities from 22 healthy volunteers and 6 MT patients at rest time by adrenaline (AD) and isoproterenol (IP). (PDF)

S5 Table The raw data of adenylate cyclase activities from 12 healthy volunteers and 11 VT patients at HUT test by adrenaline (AD) and isoproterenol (IP). (PDF)

S6 Table The raw data of adenylate cyclase activities from 12 healthy volunteers and 6 MT patients at HUT test by adrenaline (AD) and isoproterenol (IP). (PDF)

S7 Table The raw data of adenylate cyclase activities in the different seasons from three volunteers by adrenaline (AD) and isoproterenol (IP). Upper: Adrenaline
Lower: Isoproterenol.
(PDF)

S8 Table The raw data of adenylate cyclase activities in the different seasons from C41 volunteer by adrenaline (AD) and isoproterenol (IP). The AC activities in the different seasons from the healthy volunteer during the HUT test. HUT was tested in two different seasons on the 15^th^ of July and the 29^th^ of September.
Upper: Adrenaline
Lower: Isoproterenol.
(PDF)
