# Supplementary material for: Neurally mediated syncope diagnosis based on adenylate cyclase activity in Japanese patients
Source: PLoS One. 2019 Apr 18;14(4):e0214733. doi: 10.1371/journal.pone.0214733 (PMC6472876; doi:10.1371/journal.pone.0214733)
Supplement: S1 Table — (PDF) [file pone.0214733.s001.pdf]

Supplementary S1 Table. An example of the raw data of healthy volunteer (C48)

		C48-1		Baseline	C48-2		70°	C48-3		10 minutes	C48-4		20 minutes
		Average	C3-		Average			Average			Average		
BLANK	0	1609648	0		1645505			1666010			1550813		
cAMP 1.95uM	1950000	46647.3	1563001										
cAMP 781nM	781000	44777.17	1564871										
cAMP 312nM	312000	45511.92	1564136										
cAMP 125nM	125000	63706.78	1545942										
cAMP 50nM	50000	445662	1163986										
cAMP 20nM	20000	1172298	437350										
cAMP 8nM	8000	1464182	145466.7										
cAMP 3.2nM	3200	1575528	34120										
cAMP 1.28nM	1280	1561903	47745										
			C37-			G37-			K37-			O37-	
Forskolin 100uM	100000	89217.35	1612208		94675.9	1677886		92525.97	1666964		77569.02	1671330	
Forskolin 20uM	20000	320368	1381057		350258.7	1422303		474948.2	1284542		388200	1360699	
Forskolin 4uM	4000	1278022	423403.3		1367338	405223.3		1366693	392796.7		1389548	359350.8	
Forskolin 800nM	800	1639190	62235		1695252	77310		1635700	123790		1699653	49245.76	
Forskolin 160nM	160	1694570	6855		1754340	18221.67		1733573	25916.67		1774352	-25452.6	
Forskolin 32nM	32	1759188	-57763.3		1804000	-31438.3		1727340	32150		1800207	-51307.6	
				/D14			/H14			/L14			/P14
Isoproterenol 5mM	5000000	756389.7	945035.3	0.586175	798097.3	974464.3	0.580769	842956	916534	0.549822	807190	941709.1	0.563449
Isoproterenol 500uM	500000	837539	863886	0.53584	938104.8	834456.8	0.497326	1004642	754848.3	0.452828	1007083	741815.9	0.443848
Isoproterenol 50uM	50000	985475	715950	0.444081	1126712	645850	0.384919	1156723	602766.7	0.361595	1044013	704885.8	0.421751
Isoproterenol 5uM	5000	1042006	659418.7	0.409016	1271348	501213.3	0.298717	1265112	494378.3	0.296574	1222448	526450.8	0.314989
Isoproterenol 500nM	500	1302423	399001.7	0.247488	1427077	345485	0.205905	1402105	357385	0.214393	1390922	357977.4	0.214187
Isoproterenol 50nM	50	1623652	77773.33	0.04824	1651498	121063.3	0.072152	1716873	42616.67	0.025565	1744130	4769.091	0.002853
Isoproterenol 5nM	5	1743805	-42380	-0.02629	1780632	-8070	-0.00481	1757625	1865	0.001119	1838933	-90034.2	-0.05387
Adrenaline 1mM	1000000	797262.7	904162.3	0.560823	866755.5	905806.2	0.53985	873575	885915	0.531454	857698	891201.1	0.533229
Adrenaline 100uM	100000	942679.2	758745.8	0.470625	1078878	693683.3	0.413427	1153960	605530	0.363253	1115347	633552.4	0.379071
Adrenaline 10uM	10000	1101728	599696.7	0.371972	1276995	495566.7	0.295352	1259922	499568.3	0.299688	1239183	509715.8	0.304976
Adrenaline 1uM	1000	1419763	281661.7	0.174706	1516183	256378.3	0.152798	1532838	226651.7	0.135967	1507078	241820.8	0.144688
Adrenaline 100nM	100	1657878	43546.67	0.027011	1719248	53313.33	0.031774	1722903	36586.67	0.021948	1718305	30594.09	0.018305
Adrenaline 10nM	10	1656835	44590	0.027658	1743162	29400	0.017522	1724742	34748.33	0.020845	1737262	11637.42	0.006963
Adrenaline 1nM	1	1722678	-21253.3	-0.01318	1843763	-71200.8	-0.04243	1760940	-1450	-0.00087	1785720	-36820.9	-0.02203
Lymphocyte only		1701425			1772562			1759490			1748899		
