# Supplementary material for: Neurally mediated syncope diagnosis based on adenylate cyclase activity in Japanese patients
Source: PLoS One. 2019 Apr 18;14(4):e0214733. doi: 10.1371/journal.pone.0214733 (PMC6472876; doi:10.1371/journal.pone.0214733)
Supplement: S2 Table — (PDF) [file pone.0214733.s002.pdf]

S2 Table. The raw data of adenylate cyclase activities from 22 healthy volunteers and 18 NMS patients at rest time by adrenaline (AD) and isoproterenol (IP).

Healthy n=22	1	2	3	4	5	6	7	8	9	10	11	12	13	14	15	16	17	18	19	20	21	22		
	C11	C12	C15	C18	C19	C21	C22	C27	C31	C32	C34	C36	C37	C38	C40	C41	C44	C45	C46	C48	C51	C53	**Ave.**	SD
Adrenaline 1mM	0.665778	0.709712	0.605874	0.679459	0.66414	0.648605	0.659015	0.627572	0.619857	0.598856	0.643871	0.521069	0.636478	0.680594	0.698643	0.653925	0.595107	0.618414	0.642209	0.560823			**0.6365**	0.045584
Adrenaline 100uM	0.382489	0.684619	0.517828	0.671012	0.483418	0.488458	0.535409	0.266942	0.689506	0.358322	0.529993	0.761974	0.473305	0.506677	0.504345	0.458885	0.500938	0.618381	0.585582	0.470625	0.61892	0.675834	**0.53561**	0.119677
Adrenaline 10uM	0.180688	0.493032	0.450436	0.620559	0.333002	0.222251	0.275791	0.114208	0.485896	0.247573	0.446659	0.648401	0.318276	0.261932	0.259273	0.111025	0.34603	0.394816	0.283347	0.371972	0.416767	0.504962	**0.35395**	0.14584
Adrenaline 1uM	0.107534	0.320296	0.283733	0.474591	0.154205	0.096315	0.210773	0.091961	0.238154	0.078	0.264956	0.276602	0.127793	0.113756	0.127035	0.07354	0.170153	0.155721	0.139345	0.174706	0.209908	0.233382	**0.18738**	0.096448
Adrenaline 100nM	0.00513	0.032779	0.096791	0.072024	-0.01772	0.045393	0.006167	-0.0068	0.020975	0.043213	0.056215	0.004949	0.000302	-0.00944	0.049311	0.044332	0.020905	-0.00616	0.014158	0.027011			**0.02498**	0.029931
Adrenaline 10nM	0.000772	0.038757	0.064506	0.007085	-0.00368	0.001048	0.027036	0.006591	-0.02026	0.019966	0.033297	-0.01609	-0.00296	0.035078	0.047313	0.020914	0.004542	-0.01043	0.003003	0.027658			**0.01421**	0.022315
Adrenaline 1nM	0.019697	-0.00545	0.036996	-1.2E-05	0.030544	-0.00934	0.036254	-0.00643	-0.02535	0.009534	0.027782	-0.03253	-0.01143	0.011143	0.010164	0.008347	-0.00524	-0.02445	0.006562	-0.01318			**0.00318**	0.020253
	C11	C12	C15	C18	C19	C21	C22	C27	C31	C32	C34	C36	C37	C38	C40	C41	C44	C45	C46	C48	C51	C53		
Isoproterenol 5mM	0.669095	0.703023	0.623355	0.690352	0.673022	0.699203	0.708399	0.675712	0.69715	0.710542	0.703129	0.575502	0.66463	0.695884	0.692927	0.678168	0.611632	0.652262	0.66928	0.586175			**0.66897**	0.040051
Isoproterenol 500uM	0.608009	0.757064	0.566149	0.687068	0.681287	0.630683	0.681743	0.517073	0.69555	0.681416	0.736972	0.685033	0.545955	0.627609	0.598349	0.549981	0.537974	0.685699	0.621838	0.53584			**0.63156**	0.071952
Isoproterenol 50uM	0.288006	0.546953	0.487765	0.651297	0.468436	0.3163	0.428554	0.180695	0.616843	0.362266	0.492994	0.765834	0.441879	0.351528	0.339658	0.216537	0.430339	0.513744	0.363962	0.444081	0.45709	0.590449	**0.44342**	0.140251
Isoproterenol 5uM	0.188182	0.496038	0.440036	0.658714	0.327386	0.204281	0.262864	0.12156	0.490538	0.277363	0.4665	0.640779	0.356776	0.255737	0.26817	0.113567	0.362864	0.43042	0.274708	0.409016	0.436873	0.527037	**0.36406**	0.150783
Isoproterenol 500nM	0.123776	0.362219	0.331471	0.52107	0.203149	0.135653	0.22689	0.093842	0.329655	0.167712	0.343476	0.358901	0.149965	0.193664	0.172408	0.10231	0.212486	0.276707	0.196201	0.247488	0.295521	0.395715	**0.24729**	0.109484
Isoproterenol 50nM	0.037773	0.07921	0.117023	0.071785	-0.02106	0.045873	0.008457	0.01532	0.021355	0.018697	0.050615	-0.00447	-0.03872	0.05684	0.102597	0.030603	-0.00243	0.001456	0.010148	0.04824			**0.03247**	0.039961
Isoproterenol 5nM	0.010342	0.007737	0.026485	0.008271	-0.0284	0.023353	-0.01194	-0.02223	-0.01318	-0.04152	-0.00162	-0.02807	-0.02625	-0.04086	-0.00476	0.021786	-0.0364	-0.05255	-0.00086	-0.02629			**-0.01185**	0.023441

NMS n=18	1	2	3	4	5	6	7	8	9	10	11	12	13	14	15	16	17	18			
	S006	S010	S011	S012	S013	S014	S018	S020	S022	S023	S025	S027	S028	S030	S033	S038	S039	S040	**Ave.**	SD	T-test
Adrenaline 1mM	0.685624	0.487138	0.750893	0.602188	0.703524	0.609078	0.701958	0.586864	0.672472	0.547432									**0.63472**	0.081767	0.47496
Adrenaline 100uM	0.426003	0.684176	0.827716	0.477584	0.546374	0.459416	0.572592	0.60253	0.748171	0.425863	0.650868	0.567394	0.742349	0.653562	0.704688	0.431794	0.553276	0.498887	**0.5874**	0.122585	0.093738
Adrenaline 10uM	0.256626	0.676036	0.696738	0.251743	0.307234	0.294726	0.462099	0.619195	0.617245	0.206934	0.380325	0.333458	0.29156	0.364902	0.546487	0.202504	0.184153	0.262383	**0.38635**	0.172062	0.265172
Adrenaline 1uM	0.128737	0.2505	0.46025	0.119948	0.161568	0.172037	0.25945	0.355847	0.452598	0.098696	0.17769	0.07432	0.023519	0.250302	0.206664	0.11138	0.047194	0.063337	**0.18967**	0.129226	0.475421
Adrenaline 100nM	0.002387	0.014842	0.035339	-0.0088	0.009609	0.041574	0.021223	0.071953	0.023457	0.013837									**0.02254**	0.022756	0.403257
Adrenaline 10nM	0.035226	0.002706	0.012973	-0.01817	-0.02039	0.043742	0.019091	0.020693	-0.00712	0.012973									**0.01017**	0.02122	0.317463
Adrenaline 1nM	-0.01404	0.000192	0.021921	-0.04184	-0.03327	-0.00215	-0.02117	0.022942	-0.01712	-0.01919									**-0.01037**	0.021343	0.056738
	S006	S010	S011	S012	S013	S014	S018	S020	S022	S023	S025	S027	S028	S030	S033	S038	S039	S040	**Ave.**	SD	T-test
Isoproterenol 5mM	0.725266	0.568767	0.702749	0.629416	0.699681	0.640418	0.718965	0.628075	0.686357	0.588653									**0.65883**	0.055349	0.307124
Isoproterenol 500uM	0.637291	0.586099	0.835331	0.559633	0.635825	0.53767	0.661661	0.559142	0.695396	0.517951									**0.6226**	0.094257	0.397501
Isoproterenol 50uM	0.341031	0.733721	0.769463	0.367988	0.386541	0.353047	0.492343	0.621584	0.732965	0.326346	0.420591	0.570836	0.438792	0.457682	0.632723	0.293867	0.196832	0.239483	**0.46532**	0.174689	0.334851
Isoproterenol 5uM	0.265173	0.621524	0.738501	0.292338	0.294398	0.311931	0.451198	0.595527	0.652359	0.228663	0.340368	0.346525	0.266383	0.396637	0.544286	0.220749	0.145699	0.175552	**0.38266**	0.177082	0.363184
Isoproterenol 500nM	0.109187	0.296127	0.533742	0.179299	0.213354	0.188547	0.366337	0.48135	0.559745	0.143998	0.290534	0.063594	0.172424	0.259442	0.396763	0.168341	0.135114	0.16402	**0.26233**	0.148597	0.361621
Isoproterenol 50nM	-0.00523	-0.02922	0.057122	-0.04658	0.044483	0.050312	0.113573	0.090342	0.07349	0.035112									**0.03834**	0.051444	0.378051
Isoproterenol 5nM	-0.01767	-0.0404	-0.02934	-0.02739	-0.03118	0.011821	0.0196	0.061009	-0.0191	-0.03079									**-0.01035**	0.031653	0.448039
