# Supplementary material for: Neurally mediated syncope diagnosis based on adenylate cyclase activity in Japanese patients
Source: PLoS One. 2019 Apr 18;14(4):e0214733. doi: 10.1371/journal.pone.0214733 (PMC6472876; doi:10.1371/journal.pone.0214733)
Supplement: S4 Table — (PDF) [file pone.0214733.s004.pdf]

S4 Table. The raw data of adenylate cyclase activities from 22 healthy volunteers and 6 MT patients at rest time by adrenaline (AD) and isoproterenol (IP).

NMS (MT) n=6		1	2	3	4	5	6			
Rest time		S012-1	S013-1	S014-1	15S023-1	15S039	15S040	**Ave.**	**SD**	T-test
Adrenaline 1mM	1000000	0.602188	0.703524	0.609078	0.547432			**0.615555**	**0.064807**	
Adrenaline 100uM	100000	0.477584	0.546374	0.459416	0.425863	0.553276	0.498887	**0.493567**	**0.049775**	0.067354
Adrenaline 10uM	10000	0.251743	0.307234	0.294726	0.206934	0.184153	0.262383	**0.251196**	**0.048196**	0.017268
Adrenaline 1uM	1000	0.119948	0.161568	0.172037	0.098696	0.047194	0.063337	**0.110463**	**0.050706**	0.044136
Adrenaline 100nM	100	-0.0088	0.009609	0.041574	0.013837			**0.014056**	**0.020811**	
Adrenaline 10nM	10	-0.01817	-0.02039	0.043742	0.012973			**0.004538**	**0.03025**	
Adrenaline 1nM	1	-0.04184	-0.03327	-0.00215	-0.01919			**-0.02411**	**0.017366**	

Healthy volunteers n=22		1	2	3	4	5	6	7	8	9	10	11	12	13	14	15	16	17	18	19	20	21	22		
Rest time		c11	c12	c15	c18	c19-1	c21	c22	c27	C48	C31	C32	C34	C36-1	C37-1	C38-1	C40-1	C41-1	C44	C45	C46-1	C51	C53	**Ave.**	SD
Adrenaline 1mM	1000000	0.665778	0.709712	0.605874	0.679459	0.66414	0.648605	0.659015	0.627572	0.560823	0.619857	0.598856	0.643871	0.521069	0.636478	0.680594	0.698643	0.653925	0.595107	0.618414	0.642209			**0.6365**	0.045584
Adrenaline 100uM	100000	0.382489	0.684619	0.517828	0.671012	0.483418	0.488458	0.535409	0.266942	0.470625	0.689506	0.358322	0.529993	0.761974	0.473305	0.506677	0.504345	0.458885	0.500938	0.618381	0.585582	0.61892	0.675834	**0.535612**	0.119677
Adrenaline 10uM	10000	0.180688	0.493032	0.450436	0.620559	0.333002	0.222251	0.275791	0.114208	0.371972	0.485896	0.247573	0.446659	0.648401	0.318276	0.261932	0.259273	0.111025	0.34603	0.394816	0.283347	0.416767	0.504962	**0.35395**	0.14584
Adrenaline 1uM	1000	0.107534	0.320296	0.283733	0.474591	0.154205	0.096315	0.210773	0.091961	0.174706	0.238154	0.078	0.264956	0.276602	0.127793	0.113756	0.127035	0.07354	0.170153	0.155721	0.139345	0.209908	0.233382	**0.187385**	0.096448
Adrenaline 100nM	100	0.00513	0.032779	0.096791	0.072024	-0.01772	0.045393	0.006167	-0.0068	0.027011	0.020975	0.043213	0.056215	0.004949	0.000302	-0.00944	0.049311	0.044332	0.020905	-0.00616	0.014158			**0.024977**	0.029931
Adrenaline 10nM	10	0.000772	0.038757	0.064506	0.007085	-0.00368	0.001048	0.027036	0.006591	0.027658	-0.02026	0.019966	0.033297	-0.01609	-0.00296	0.035078	0.047313	0.020914	0.004542	-0.01043	0.003003			**0.014208**	0.022315
Adrenaline 1nM	1	0.019697	-0.00545	0.036996	-1.2E-05	0.030544	-0.00934	0.036254	-0.00643	-0.01318	-0.02535	0.009534	0.027782	-0.03253	-0.01143	0.011143	0.010164	0.008347	-0.00524	-0.02445	0.006562			**0.00318**	0.020253

NMS (MT) n=6		1	2	3	4	5	6			
Rest time		S012-1	S013-1	S014-1	15S023-1	15S039	15S040	**Ave.**	**SD**	T-test
Isopreterenol 5mM	5000000	0.629416	0.699681	0.640418	0.588653			**0.639542**	**0.045861**	
Isopreterenol 500uM	500000	0.559633	0.635825	0.53767	0.517951			**0.562769**	**0.051593**	
Isopreterenol 50uM	50000	0.367988	0.386541	0.353047	0.326346	0.196832	0.239483	**0.311706**	**0.076293**	0.00396
Isopreterenol 5uM	5000	0.292338	0.294398	0.311931	0.228663	0.145699	0.175552	**0.24143**	**0.069333**	0.005017
Isopreterenol 500nM	500	0.179299	0.213354	0.188547	0.143998	0.135114	0.16402	**0.170722**	**0.029106**	0.003546
Isopreterenol 50nM	50	-0.04658	0.044483	0.050312	0.035112			**0.020831**	**0.045377**	
Isopreterenol 5nM	5	-0.02739	-0.03118	0.011821	-0.03079			**-0.01939**	**0.020875**	

Healthy volunteers n=22		1	2	3	4	5	6	7	8	9	10	11	12	13	14	15	16	17	18	19	20	21	22	**Ave.**	SD
Rest time		c11	c12	c15	c18	c19-1	c21	c22	c27	C48	C31	C32	C34	C36-1	C37-1	C38-1	C40-1	C41-1	C44	C45	C46-1	C51	C53		
Isoproterenol 5mM	5000000	0.669095	0.703023	0.623355	0.690352	0.673022	0.699203	0.708399	0.675712	0.586175	0.69715	0.710542	0.703129	0.575502	0.66463	0.695884	0.692927	0.678168	0.611632	0.652262	0.66928			**0.668972**	0.040051
Isoproterenol 500uM	500000	0.608009	0.757064	0.566149	0.687068	0.681287	0.630683	0.681743	0.517073	0.53584	0.69555	0.681416	0.736972	0.685033	0.545955	0.627609	0.598349	0.549981	0.537974	0.685699	0.621838			**0.631565**	0.071952
Isoproterenol 50uM	50000	0.288006	0.546953	0.487765	0.651297	0.468436	0.3163	0.428554	0.180695	0.444081	0.616843	0.362266	0.492994	0.765834	0.441879	0.351528	0.339658	0.216537	0.430339	0.513744	0.363962	0.45709	0.590449	**0.443419**	0.140251
Isoproterenol 5uM	5000	0.188182	0.496038	0.440036	0.658714	0.327386	0.204281	0.262864	0.12156	0.409016	0.490538	0.277363	0.4665	0.640779	0.356776	0.255737	0.26817	0.113567	0.362864	0.43042	0.274708	0.436873	0.527037	**0.364064**	0.150783
Isoproterenol 500nM	500	0.123776	0.362219	0.331471	0.52107	0.203149	0.135653	0.22689	0.093842	0.247488	0.329655	0.167712	0.343476	0.358901	0.149965	0.193664	0.172408	0.10231	0.212486	0.276707	0.196201	0.295521	0.395715	**0.247285**	0.109484
Isoproterenol50nM	50	0.037773	0.07921	0.117023	0.071785	-0.02106	0.045873	0.008457	0.01532	0.04824	0.021355	0.018697	0.050615	-0.00447	-0.03872	0.05684	0.102597	0.030603	-0.00243	0.001456	0.010148			**0.032466**	0.039961
Isoproterenol 5nM	5	0.010342	0.007737	0.026485	0.008271	-0.0284	0.023353	-0.01194	-0.02223	-0.02629	-0.01318	-0.04152	-0.00162	-0.02807	-0.02625	-0.04086	-0.00476	0.021786	-0.0364	-0.05255	-0.00086			**-0.01185**	0.023441
