# Supplementary material for: Neurally mediated syncope diagnosis based on adenylate cyclase activity in Japanese patients
Source: PLoS One. 2019 Apr 18;14(4):e0214733. doi: 10.1371/journal.pone.0214733 (PMC6472876; doi:10.1371/journal.pone.0214733)
Supplement: S5 Table — (PDF) [file pone.0214733.s005.pdf]

S5 Table. The raw data of adenylate cyclase activities from 12 healthy volunteers and 11 VT patients at HUT test by adrenaline (AD) and isoproterenol (IP).

NMS (VT) n=11		1	2	3	4	5	6	7	8	9	10	11		
Base		S010-1	S011-1	S020-1	S018-1	S022-1	S025-1	S027-1	S028-1	S030-1	S033-1	S038-1	Average	SD
Isoproterenol 5mM	5000000	0.568767	0.702749	0.628075	0.718965	0.686357							0.660983	
Isoproterenol 500uM	500000	0.586099	0.835331	0.559142	0.661661	0.695396							0.667526	
Isoproterenol 50uM	50000	0.733721	0.769463	0.621584	0.492343	0.732965	0.420591	0.570836	0.438792	0.457682	0.632723	0.293867	0.560415	0.152698
Isoproterenol 5uM	5000	0.621524	0.738501	0.595527	0.451198	0.652359	0.340368	0.346525	0.266383	0.396637	0.544286	0.220749	0.470369	0.170573
Isoproterenol 500nM	500	0.296127	0.533742	0.48135	0.366337	0.559745	0.290534	0.063594	0.172424	0.259442	0.396763	0.168341	0.326218	0.158884
Isoproterenol 50nM	50	-0.02922	0.057122	0.090342	0.113573	0.07349							0.061062	
Isoproterenol 5nM	5	-0.0404	-0.02934	0.061009	0.0196	-0.0191							-0.00165	
Adrenaline 1mM	1000000	0.487138	0.750893	0.586864	0.701958	0.672472							0.639865	
Adrenaline 100uM	100000	0.684176	0.827716	0.60253	0.572592	0.748171	0.650868	0.567394	0.742349	0.653562	0.704688	0.431794	0.653258	0.107929
Adrenaline 10uM	10000	0.676036	0.696738	0.619195	0.462099	0.617245	0.380325	0.333458	0.29156	0.364902	0.546487	0.202504	0.471868	0.168761
Adrenaline 1uM	1000	0.2505	0.46025	0.355847	0.25945	0.452598	0.17769	0.07432	0.023519	0.250302	0.206664	0.11138	0.238411	0.14267
Adrenaline 100nM	100	0.014842	0.035339	0.071953	0.021223	0.023457							0.033363	
Adrenaline 10nM	10	0.002706	0.012973	0.020693	0.019091	-0.00712							0.009668	
Adrenaline 1nM	1	0.000192	0.021921	0.022942	-0.02117	-0.01712							0.001353	
70°		S010-2	S011-2	S020-2	S018-2	S022-2	S025-2	S027-2	S028-2	S030-2	S033-2	S038-2		
Isoproterenol 5mM	5000000	0.567395	0.681576	0.61814	0.682051	0.666079							0.643048	
Isoproterenol 500uM	500000	0.596599	0.777989	0.531318	0.689018	0.758639							0.670713	
Isoproterenol 50uM	50000	0.679497	0.727236	0.59271	0.527635	0.698648	0.384592	0.475822	0.492902	0.37946	0.536518	0.249628	0.522241	0.148235
Isoproterenol 5uM	5000	0.614095	0.645358	0.541985	0.477269	0.607188	0.324837	0.294697	0.26714	0.344974	0.472921	0.196118	0.435144	0.156784
Isoproterenol 500nM	500	0.316829	0.522743	0.396583	0.399744	0.557719	0.235201	0.036007	0.132435	0.254846	0.272694	0.16503	0.299075	0.160797
Isoproterenol 50nM	50	0.002822	0.05555	0.103269	0.148853	0.076943							0.077487	
Isoproterenol 5nM	5	-0.01483	0.015978	-0.0062	0.02235	-0.04951							-0.00644	
Adrenaline 1mM	1000000	0.519159	0.687653	0.585581	0.618387	0.68096							0.618348	
Adrenaline 100uM	100000	0.645164	0.773656	0.554981	0.616481	0.742601	0.614422	0.516414	0.720865	0.518816	0.66297	0.43818	0.618596	0.104676
Adrenaline 10uM	10000	0.657167	0.62745	0.508129	0.44446	0.585747	0.329431	0.286579	0.318732	0.334241	0.44186	0.199594	0.430308	0.15054
Adrenaline 1uM	1000	0.260674	0.387023	0.271924	0.267841	0.405729	0.139165	0.033594	0.055566	0.121953	0.091769	0.07886	0.192191	0.13222
Adrenaline 100nM	100	0.000345	0.077247	0.050537	0.05242	0.008616							0.037833	
Adrenaline 10nM	10	-0.00232	0.028657	0.021912	-0.0108	-0.01898							0.003695	
Adrenaline 1nM	1	0.004926	0.026981	-0.00411	-0.05271	-0.02387							-0.00976	
10 min		S010-3	S011-3	S020-3	S018-3	S022-3	S025-3	S027-3	S028-3	S030-3	S033-3	S038-3		
Isoproterenol 5mM	5000000	0.504606	0.640308	0.622595	0.642863	0.61086							0.604247	
Isoproterenol 500uM	500000	0.51933	0.758241	0.515928	0.79548	0.574282							0.632652	
Isoproterenol 50uM	50000	0.743942	0.851982	0.74846	0.691033	0.720393	0.442187	0.48591	0.538029	0.370512	0.580232	0.277888	0.586415	0.180438
Isoproterenol 5uM	5000	0.719171	0.786167	0.711526	0.649533	0.647722	0.355551	0.347945	0.3955	0.242978	0.51458	0.218619	0.508118	0.204569
Isoproterenol 500nM	500	0.394464	0.624263	0.539591	0.501392	0.4531	0.246001	0.064996	0.276522	0.081793	0.337905	0.161485	0.334683	0.186861
Isoproterenol 50nM	50	0.030549	0.121082	0.027205	0.080389	0.022713							0.056388	
Isoproterenol 5nM	5	-0.00318	0.076558	-0.03857	-0.03343	-0.01341							-0.00241	
Adrenaline 1mM	1000000	0.50789	0.677842	0.594521	0.575614	0.616314							0.594436	
Adrenaline 100uM	100000	0.692489	0.85246	0.513948	0.760478	0.670876	0.666506	0.501271	0.789848	0.657763	0.697239	1.201654	0.727685	0.188713
Adrenaline 10uM	10000	0.678469	0.75921	0.691409	0.648548	0.645979	0.378521	0.340734	0.419322	0.289854	0.469217	0.80867	0.557267	0.181659
Adrenaline 1uM	1000	0.378033	0.559693	0.38548	0.341349	0.365023	0.138492	0.029099	0.149118	-0.00617	0.17563	0.302689	0.256221	0.17238
Adrenaline 100nM	100	0.003833	0.123474	0.014661	0.00831	0.008865							0.031829	
Adrenaline 10nM	10	-0.01047	0.099127	-0.00081	-0.03207	-0.02395							0.006365	
Adrenaline 1nM	1	-0.03997	0.06751	-0.03233	-0.04748	-0.03276							-0.01701	
20 min		S010-4	S011-4	S020-4	S018-4	S022-4	S025-4	S027-4	S028-4	S030-4	S033-4	S038-4		
Isoproterenol 5mM	5000000	0.496711	0.615417	0.60925	0.641134	0.650141							0.602531	
Isoproterenol 500uM	500000	0.483746	0.714936	0.476465	0.725084	0.570216							0.594089	
Isoproterenol 50uM	50000	0.726675	0.851575	0.467004	0.564148	0.583106	0.555561	0.46789	0.943602	0.425595	0.798589	0.383465	0.615201	0.187159
Isoproterenol 5uM	5000	0.663717	0.787797	0.635865	0.526052	0.528271	0.42243	0.305321	0.823986	0.237546	0.740131	0.3006	0.542883	0.206041
Isoproterenol 500nM	500	0.348455	0.570592	0.104036	0.363326	0.341548	0.298335	0.076658	0.605585	0.123164	0.395797	0.232379	0.314534	0.174834
Isoproterenol 50nM	50	0.023837	0.010731	-0.04001	0.016022	-0.01069							-2.3E-05	
Isoproterenol 5nM	5	-0.00582	-0.0156	-0.05977	-0.05355	-0.0474							-0.03643	
Adrenaline 1mM	1000000	0.516423	0.684465	0.570804	0.56563	0.671085							0.601681	
Adrenaline 100uM	100000	0.657282	0.862138	0.425649	0.652369	0.558295	0.675027	0.507124	1.034919	0.624778	0.869849	0.496692	0.669466	0.184746
Adrenaline 10uM	10000	0.670621	0.79486	0.657591	0.497933	0.535603	0.369575	0.31338	0.847719	0.286675	0.698028	0.31616	0.544377	0.20292
Adrenaline 1uM	1000	0.37494	0.612562	0.204264	0.245087	0.312681	0.11157	0.039975	0.304423	0.069236	0.239623	0.163045	0.243401	0.160999
Adrenaline 100nM	100	0.041646	0.135315	-0.01526	0.021547	0.055079							0.047665	
Adrenaline 10nM	10	0.04105	0.069168	-0.03001	0.007554	0.026889							0.022931	
Adrenaline 1nM	1	0.041774	0.028757	0.013725	0.003925	0.005555							0.018747	

Healthy n=12		1	2	3	4	5	6	7	8	9	10	11	12			
Base		C36-1	C37-1	C38-1	C040-1	C41-1	C43-1	C46-1	C47-1	C048-1	C050-1	C051-1	C053-1	Average	SD	T-test
Isoproterenol 5mM	5000000	0.575502	0.66463	0.695884	0.692927	0.678168	0.616207	0.66928	0.626701	0.586175	0	0		0.633191		
Isoproterenol 500uM	500000	0.685033	0.545955	0.627609	0.598349	0.549981	0.486665	0.621838	0.556789	0.53584	0	0		0.571728		
Isoproterenol 50uM	50000	0.765834	0.441879	0.351528	0.339658	0.216537	0.378901	0.363962	0.275046	0.444081	0.232386	0.45709	0.590449	0.404779	0.154299	0.01212
Isoproterenol 5uM	5000	0.640779	0.356776	0.255737	0.26817	0.113567	0.353159	0.274708	0.20157	0.409016	0.125952	0.436873	0.527037	0.330279	0.15716	0.027111
Isoproterenol 500nM	500	0.358901	0.149965	0.193664	0.172408	0.10231	0.261852	0.196201	0.160736	0.247488	0.140866	0.295521	0.395715	0.222969	0.090619	0.038632
Isoproterenol 50nM	50	-0.00447	-0.03872	0.05684	0.102597	0.030603	0.050547	0.010148	0.041667	0.04824	0	0		0.027011		
Isoproterenol 5nM	5	-0.02807	-0.02625	-0.04086	-0.00476	0.021786	-0.00598	-0.00086	0.004312	-0.02629	0	0		-0.01428		
Adrenaline 1mM	1000000	0.521069	0.636478	0.680594	0.698643	0.653925	0.615496	0.642209	0.57025	0.560823	0	0		0.60463		
Adrenaline 100uM	100000	0.761974	0.473305	0.506677	0.504345	0.458885	0.484882	0.585582	0.488452	0.470625	0.464659	0.61892	0.675834	0.541178	0.097983	0.008511
Adrenaline 10uM	10000	0.648401	0.318276	0.261932	0.259273	0.111025	0.379446	0.283347	0.204512	0.371972	0.163163	0.416767	0.504962	0.326923	0.149877	0.021068
Adrenaline 1uM	1000	0.276602	0.127793	0.113756	0.127035	0.07354	0.359127	0.139345	0.097657	0.174706	0.0945	0.209908	0.233382	0.168946	0.085663	0.090243
Adrenaline 100nM	100	0.004949	0.000302	-0.00944	0.049311	0.044332	0.265821	0.014158	0.017002	0.027011	0	0		0.043461		
Adrenaline 10nM	10	-0.01609	-0.00296	0.035078	0.047313	0.020914	0.051841	0.003003	0.005244	0.027658	0	0		0.016927		
Adrenaline 1nM	1	-0.03253	-0.01143	0.011143	0.010164	0.008347	-0.01477	0.006562	-0.01425	-0.01318	0	0		-0.00814		
70°		C36-2	C37-2	C38-2	C040-2	C41-2	C43-2	C46-2	C47-2	C048-2	C050-2	C051-2	C053-2			
Isoproterenol 5mM	5000000	0.540608	0.610219	0.701339	0.675345	0.65798	0.585035	0.641233	0.6516	0.580769	0	0		0.616617		
Isoproterenol 500uM	500000	0.66948	0.501615	0.604967	0.628939	0.519893	0.436723	0.532393	0.677576	0.497326	0	0		0.548589		
Isoproterenol 50uM	50000	0.701006	0.38992	0.277178	0.370136	0.164946	0.357192	0.276163	0.412235	0.384919	0.162713	0.457463	0.590068	0.378662	0.156597	0.017344
Isoproterenol 5uM	5000	0.605642	0.309221	0.199197	0.299357	0.111925	0.257048	0.161475	0.292475	0.298717	0.128747	0.460466	0.531201	0.304623	0.156206	0.029483
Isoproterenol 500nM	500	0.356867	0.145321	0.167315	0.232847	0.054602	0.169873	0.088058	0.200724	0.205905	0.108746	0.302144	0.388832	0.201769	0.103897	0.053093
Isoproterenol 50nM	50	0.013634	-0.01311	0.10221	0.124296	0.026071	-0.06476	-0.02964	0.061021	0.072152	0	0		0.026636		
Isoproterenol 5nM	5	-0.01947	-0.02522	0.020029	0.036247	-0.00439	-0.14128	-0.04897	0.014085	-0.00481	0	0		-0.02387		
Adrenaline 1mM	1000000	0.495798	0.569605	0.704251	0.678227	0.631304	0.532862	0.604557	0.585728	0.53985	0	0		0.5782		
Adrenaline 100uM	100000	0.705793	0.417502	0.453322	0.526494	0.412419	0.357069	0.460932	0.643443	0.413427	0.418743	0.579005	0.68229	0.50587	0.118856	0.012396
Adrenaline 10uM	10000	0.615054	0.278172	0.188715	0.265434	0.104945	0.232808	0.176941	0.309194	0.295352	0.143949	0.37588	0.465157	0.287633	0.143551	0.015306
Adrenaline 1uM	1000	0.289071	0.109531	0.118544	0.143075	0.072936	0.041349	0.07664	0.153219	0.152798	0.107439	0.157874	0.150286	0.131063	0.062326	0.092026
Adrenaline 100nM	100	0.023548	0.011804	0.022636	0.031326	0.028094	-0.12746	-0.0394	0.025233	0.031774	0	0		0.000889		
Adrenaline 10nM	10	0.014744	0.001621	0.014448	0.012697	0.026009	-0.12534	-0.00858	0.003152	0.017522	0	0		-0.00432		
Adrenaline 1nM	1	0.00012	-0.02532	0.033895	-0.02517	0.00936	-0.139	-0.02015	0.011234	-0.04243	0	0		-0.02386		
10 min		C36-3	C37-3	C38-3	C040-3	C41-3	C43-3	C46-3	C47-3	C048-3	C050-3	C051-3	C053-3			
Isoproterenol 5mM	5000000	0.469615	0.605519	0.638618	0.658659	0.651187	0.58504	0.602827	0.586696	0.549822	0	0		0.582127		
Isoproterenol 500uM	500000	0.505705	0.505436	0.600133	0.611908	0.531388	0.428744	0.455444	0.56219	0.452828	0	0		0.499411		
Isoproterenol 50uM	50000	0.69008	0.427835	0.363097	0.506611	0.287074	0.30101	0.238809	0.4337	0.361595	0.22787	0.399463	0.520767	0.396493	0.132411	0.00519
Isoproterenol 5uM	5000	0.573857	0.357813	0.289166	0.421053	0.13344	0.240197	0.138893	0.274154	0.296574	0.18943	0.355666	0.481964	0.312684	0.134018	0.007836
Isoproterenol 500nM	500	0.3233	0.146472	0.233869	0.241755	0.083287	0.173743	0.088176	0.167332	0.214393	0.147499	0.268123	0.336092	0.202004	0.082528	0.024236
Isoproterenol 50nM	50	-0.02209	0.020922	0.069656	0.036547	0.031046	0.013052	-0.00972	0.021185	0.025565	0	0		0.019464		
Isoproterenol 5nM	5	-0.02169	0.001455	0.015022	-0.00883	0.011616	-0.02416	-0.01661	-0.03351	0.001119	0	0		-0.00729		
Adrenaline 1mM	1000000	0.438058	0.588412	0.654646	0.651939	0.621838	0.545607	0.56437	0.541464	0.531454	0	0		0.557478		
Adrenaline 100uM	100000	0.577236	0.440056	0.519117	0.607661	0.446216	0.341682	0.390337	0.602928	0.363253	0.48952	0.535941	0.63866	0.496051	0.100559	0.001249
Adrenaline 10uM	10000	0.520062	0.337168	0.263434	0.381105	0.163302	0.205381	0.147584	0.273239	0.299688	0.203486	0.31466	0.446966	0.296339	0.112927	0.000402
Adrenaline 1uM	1000	0.240591	0.118109	0.134269	0.188301	0.076393	0.111531	0.061781	0.114963	0.135967	0.109947	0.108816	0.183192	0.131988	0.050047	0.02039
Adrenaline 100nM	100	-0.00672	0.023303	0.009842	0.030431	0.040758	-0.00826	0.005414	0.006605	0.021948	0	0		0.01276		
Adrenaline 10nM	10	-0.00639	0.010954	0.003866	0.021449	0.040138	-0.01987	-0.00834	-0.00039	0.020845	0	0		0.006851		
Adrenaline 1nM	1	-0.04256	0.002028	0.01005	0.008151	0.031315	-0.00721	-0.02067	-0.01	-0.00087	0	0		-0.00431		
20 min		C36-4	C37-4	C38-4	C040-4	C41-4	C43-4	C46-4	C47-4	C048-4	C050-4	C051-4	C053-4	Average		
Isoproterenol 5mM	5000000	0.48141	0.59351	0.6557	0.642971	0.629867	0.568406	0.59554	0.552801	0.563449	0	0	0	0.578237		
Isoproterenol 500uM	500000	0.487475	0.517069	0.651278	0.57275	0.533343	0.423929	0.444228	0.527197	0.443848	0	0	0	0.496913		
Isoproterenol 50uM	50000	0.629321	0.612423	0.449734	0.403493	0.298672	0.319218	0.26333	0.4659	0.421751	0.279892	0.469228	0.464759	0.423144	0.119647	0.005006
Isoproterenol 5uM	5000	0.550668	0.548669	0.333733	0.322789	0.140863	0.281765	0.157021	0.308233	0.314989	0.2479	0.392549	0.375606	0.331232	0.127212	0.004782
Isoproterenol 500nM	500	0.259487	0.222269	0.310466	0.177412	0.069729	0.192632	0.066295	0.220416	0.214187	0.17775	0.273197	0.278378	0.205185	0.076242	0.038579
Isoproterenol 50nM	50	-0.01954	-0.02067	0.031378	0.047046	-0.00315	0.004184	-0.06001	0.017568	0.002853	0	0	0	-0.00495		
Isoproterenol 5nM	5	-0.02733	-0.04237	-0.06312	-0.01285	0.005228	-0.03773	-0.05439	-0.09231	-0.05387	0	0	0	-0.04664		
Adrenaline 1mM	1000000	0.463035	0.57392	0.680409	0.654641	0.64785	0.5609	0.575303	0.54353	0.533229	0	0	0	0.567934		
Adrenaline 100uM	100000	0.576932	0.51415	0.633898	0.547847	0.467596	0.370675	0.416147	0.619397	0.379071	0.49629	0.572353	0.5781	0.514371	0.089895	0.012058
Adrenaline 10uM	10000	0.571906	0.562986	0.336593	0.277317	0.201285	0.276292	0.171319	0.309766	0.304976	0.222378	0.402894	0.370702	0.334034	0.127794	0.004623
Adrenaline 1uM	1000	0.236336	0.199332	0.147867	0.138014	0.11307	0.143043	0.071892	0.159119	0.144688	0.123798	0.129612	0.128095	0.144572	0.041439	0.03659
Adrenaline 100nM	100	0.032552	-0.00636	0.019104	0.015082	0.053104	0.0114	-0.01779	0.027575	0.018305	0	0	0	0.017355		
Adrenaline 10nM	10	-0.01803	0.00832	0.009432	0.025275	0.052211	-0.00486	-0.01361	0.039907	0.006963	0	0	0	0.009699		
Adrenaline 1nM	1	-0.0102	-0.01384	0.014224	0.004534	0.037719	-0.00633	-0.03471	0.040481	-0.02203	0	0	0	-0.00186		
