# Supplementary material for: Neurally mediated syncope diagnosis based on adenylate cyclase activity in Japanese patients
Source: PLoS One. 2019 Apr 18;14(4):e0214733. doi: 10.1371/journal.pone.0214733 (PMC6472876; doi:10.1371/journal.pone.0214733)
Supplement: S6 Table — (PDF) [file pone.0214733.s006.pdf]

S6 Table. The raw data of adenylate cyclase activities from 12 healthy volunteers and 6 MT patients at HUT test by adrenaline (AD) and isoproterenol (IP).

NMS n=6

Base

		1	2	3	4	5	6		
		S012-1	S013-1	S014-1	S023-1	S039-1	S040-1	Ave.	SD
Isoproterenol 5mM	5000000	0.629416	0.699681	0.640418	0.588653			0.639542	0.045861
Isoproterenol 500uM	500000	0.559633	0.635825	0.53767	0.517951			0.562769	0.051593
Isoproterenol 50uM	50000	0.367988	0.386541	0.353047	0.326346	0.196832	0.239483	0.311706	0.076293
Isoproterenol 5uM	5000	0.292338	0.294398	0.311931	0.228663	0.145699	0.175552	0.24143	0.069333
Isoproterenol 500nM	500	0.179299	0.213354	0.188547	0.143998	0.135114	0.16402	0.170722	0.029106
Isoproterenol 50nM	50	-0.04658	0.044483	0.050312	0.035112			0.020831	0.045377
Isoproterenol 5nM	5	-0.02739	-0.03118	0.011821	-0.03079			-0.01939	0.020875
Adrenaline 1mM	1000000	0.602188	0.703524	0.609078	0.547432			0.615555	0.064807
Adrenaline 100uM	100000	0.477584	0.546374	0.459416	0.425863	0.553276	0.498887	0.493567	0.049775
Adrenaline 10uM	10000	0.251743	0.307234	0.294726	0.206934	0.184153	0.262383	0.251196	0.048196
Adrenaline 1uM	1000	0.119948	0.161568	0.172037	0.098696	0.047194	0.063337	0.110463	0.050706
Adrenaline 100nM	100	-0.0088	0.009609	0.041574	0.013837			0.014056	0.020811
Adrenaline 10nM	10	-0.01817	-0.02039	0.043742	0.012973			0.004538	0.03025
Adrenaline 1nM	1	-0.04184	-0.03327	-0.00215	-0.01919			-0.02411	0.017366

Healthy n=12

Base

		1	2	3	4	5	6	7	8	9	10	11	12			
		C36-1	C37-1	C38-1	C040-1	C41-1	C43-1	C46-1	C47-1	C048-1	C050-1	C051-1	C053-1	Average	SD	T-test
Isoproterenol 5mM	5000000	0.575502	0.66463	0.695884	0.692927	0.678168	0.616207	0.66928	0.626701	0.586175	0	0		0.52777		
Isoproterenol 500uM	500000	0.685033	0.545955	0.627609	0.598349	0.549981	0.486665	0.621838	0.556789	0.53584	0	0		0.47346		
Isoproterenol 50uM	50000	0.765834	0.441879	0.351528	0.339658	0.216537	0.378901	0.363962	0.275046	0.444081	0.232386	0.45709	0.590449	0.404779	0.154299	0.053075
Isoproterenol 5uM	5000	0.640779	0.356776	0.255737	0.26817	0.113567	0.353159	0.274708	0.20157	0.409016	0.125952	0.436873	0.527037	0.330279	0.15716	0.058083
Isoproterenol 500nM	500	0.358901	0.149965	0.193664	0.172408	0.10231	0.261852	0.196201	0.160736	0.247488	0.140866	0.295521	0.395715	0.222969	0.090619	0.044758
Isoproterenol 50nM	50	-0.00447	-0.03872	0.05684	0.102597	0.030603	0.050547	0.010148	0.041667	0.04824	0	0		0.027041		
Isoproterenol 5nM	5	-0.02807	-0.02625	-0.04086	-0.00476	0.021786	-0.00598	-0.00086	0.004312	-0.02629	0	0		-0.00972		
Adrenaline 1mM	1000000	0.521069	0.636478	0.680594	0.698643	0.653925	0.615496	0.642209	0.57025	0.560823	0	0		0.507226		
Adrenaline 100uM	100000	0.761974	0.473305	0.506677	0.504345	0.458885	0.484882	0.585582	0.488452	0.470625	0.464659	0.61892	0.675834	0.541178	0.097983	0.095287
Adrenaline 10uM	10000	0.648401	0.318276	0.261932	0.259273	0.111025	0.379446	0.283347	0.204512	0.371972	0.163163	0.416767	0.504962	0.326923	0.149877	0.066226
Adrenaline 1uM	1000	0.276602	0.127793	0.113756	0.127035	0.07354	0.359127	0.139345	0.097657	0.174706	0.0945	0.209908	0.233382	0.168946	0.085663	0.044716
Adrenaline 100nM	100	0.004949	0.000302	-0.00944	0.049311	0.044332	0.265821	0.014158	0.017002	0.027011	0	0		0.037586		
Adrenaline 10nM	10	-0.01609	-0.00296	0.035078	0.047313	0.020914	0.051841	0.003003	0.005244	0.027658	0	0		0.015636		
Adrenaline 1nM	1	-0.03253	-0.01143	0.011143	0.010164	0.008347	-0.01477	0.006562	-0.01425	-0.01318	0	0		-0.00454		

70°

		1	2	3	4	5	6		
		S012-2	S013-2	S014-2	S023-2	S039-2	S040-2	Ave.	SD
Isoproterenol 5mM	5000000	0.635191	0.700375	0.631188	0.58767			0.638606	0.046464
Isoproterenol 500uM	500000	0.589408	0.64624	0.519012	0.552729			0.576847	0.054466
Isoproterenol 50uM	50000	0.390474	0.382622	0.311407	0.317287	0.156795	0.223733	0.297053	0.091338
Isoproterenol 5uM	5000	0.312679	0.272516	0.257444	0.231851	0.073495	0.156341	0.217388	0.087596
Isoproterenol 500nM	500	0.215852	0.177094	0.136247	0.170848	0.084296	0.147212	0.155258	0.044392
Isoproterenol 50nM	50	0.010102	0.024513	-0.01777	0.055617			0.018115	0.030548
Isoproterenol 5nM	5	-0.03631	-0.0225	-0.0124	0.030218			-0.01025	0.028703
Adrenaline 1mM	1000000	0.618843	0.710809	0.584996	0.562071			0.61918	0.065386
Adrenaline 100uM	100000	0.48837	0.529095	0.407421	0.434569	0.408176	0.452572	0.453367	0.04789
Adrenaline 10uM	10000	0.289032	0.283581	0.241425	0.229432	0.108052	0.177597	0.22152	0.068808
Adrenaline 1uM	1000	0.155253	0.137498	0.100439	0.120503	0.013378	-0.00822	0.086475	0.067819
Adrenaline 100nM	100	0.004206	-0.00584	0.001725	0.026291			0.006595	0.013809
Adrenaline 10nM	10	-0.0198	0.007868	-0.00845	0.04204			0.005416	0.026927
Adrenaline 1nM	1	-0.0343	-0.01639	0.000838	-0.00435			-0.01355	0.015601

70°		C36-2	C37-2	C38-2	C040-2	C41-2	C43-2	C46-2	C47-2	C048-2	C050-2	C051-2	C053-2	Average	SD	T-test
Isoproterenol 5mM	5000000	0.540608	0.610219	0.701339	0.675345	0.65798	0.585035	0.641233	0.6516	0.580769	0	0		0.513102		
Isoproterenol 500uM	500000	0.66948	0.501615	0.604967	0.628939	0.519893	0.436723	0.532393	0.677576	0.497326	0	0		0.46081		
Isoproterenol 50uM	50000	0.701006	0.38992	0.277178	0.370136	0.164946	0.357192	0.276163	0.412235	0.384919	0.162713	0.457463	0.590068	0.378662	0.156597	0.091763
Isoproterenol 5uM	5000	0.605642	0.309221	0.199197	0.299357	0.111925	0.257048	0.161475	0.292475	0.298717	0.128747	0.460466	0.531201	0.304623	0.156206	0.074785
Isoproterenol 500nM	500	0.356867	0.145321	0.167315	0.232847	0.054602	0.169873	0.088058	0.200724	0.205905	0.108746	0.302144	0.388832	0.201769	0.103897	0.101609
Isoproterenol 50nM	50	0.013634	-0.01311	0.10221	0.124296	0.026071	-0.06476	-0.02964	0.061021	0.072152	0	0		0.026534		
Isoproterenol 5nM	5	-0.01947	-0.02522	0.020029	0.036247	-0.00439	-0.14128	-0.04897	0.014085	-0.00481	0	0		-0.0158		
Adrenaline 1mM	1000000	0.495798	0.569605	0.704251	0.678227	0.631304	0.532862	0.604557	0.585728	0.53985	0	0		0.485653		
Adrenaline 100uM	100000	0.705793	0.417502	0.453322	0.526494	0.412419	0.357069	0.460932	0.643443	0.413427	0.418743	0.579005	0.68229	0.50587	0.118856	0.101355
Adrenaline 10uM	10000	0.615054	0.278172	0.188715	0.265434	0.104945	0.232808	0.176941	0.309194	0.295352	0.143949	0.37588	0.465157	0.287633	0.143551	0.102607
Adrenaline 1uM	1000	0.289071	0.109531	0.118544	0.143075	0.072936	0.041349	0.07664	0.153219	0.152798	0.107439	0.157874	0.150286	0.131063	0.062326	0.104326
Adrenaline 100nM	100	0.023548	0.011804	0.022636	0.031326	0.028094	-0.12746	-0.0394	0.025233	0.031774	0	0		0.000687		
Adrenaline 10nM	10	0.014744	0.001621	0.014448	0.012697	0.026009	-0.12534	-0.00858	0.003152	0.017522	0	0		-0.00397		
Adrenaline 1nM	1	0.00012	-0.02532	0.033895	-0.02517	0.00936	-0.139	-0.02015	0.011234	-0.04243	0	0		-0.01795		

10 min

		1	2	3	4	5	6		
		S012-3	S013-3	S014-3	S023-3	S039-3	S040-3	Ave.	SD
Isoproterenol 5mM	5000000	0.650323	0.653414	0.596605	0.565789			0.616533	0.042716
Isoproterenol 500uM	500000	0.677198	0.626104	0.508777	0.441475			0.563388	0.107596
Isoproterenol 50uM	50000	0.545272	0.527306	0.499441	0.319967	0.171468	0.600625	0.444013	0.163987
Isoproterenol 5uM	5000	0.506127	0.377846	0.482369	0.178227	0.088993	0.596314	0.371646	0.199079
Isoproterenol 500nM	500	0.267799	0.233831	0.239514	0.056473	0.053766	0.563559	0.235824	0.18642
Isoproterenol 50nM	50	0.000294	0.015673	0.05046	-0.10624			-0.00995	0.067532
Isoproterenol 5nM	5	0.030992	-0.02113	0.004519	-0.11867			-0.02607	0.065298
Adrenaline 1mM	1000000	0.63268	0.688006	0.599002	0.558481			0.619542	0.054803
Adrenaline 100uM	100000	0.635883	0.609969	0.483399	0.439029	0.393529	0.767916	0.554954	0.1411
Adrenaline 10uM	10000	0.432089	0.432133	0.454645	0.213408	0.093902	0.597713	0.370648	0.18302
Adrenaline 1uM	1000	0.22527	0.206351	0.233232	0.012479	-0.00236	0.27425	0.158204	0.12077
Adrenaline 100nM	100	0.009144	0.033766	0.037635	-0.13206			-0.01288	0.080449
Adrenaline 10nM	10	0.046842	-0.04121	0.049339	-0.1102			-0.01381	0.07683
Adrenaline 1nM	1	0.074276	-0.09217	-0.00754	-0.16501			-0.04761	0.10365

10 min		C36-3	C37-3	C38-3	C040-3	C41-3	C43-3	C46-3	C47-3	C048-3	C050-3	C051-3	C053-3	Average	SD	T-test
Isoproterenol 5mM	5000000	0.469615	0.605519	0.638618	0.658659	0.651187	0.58504	0.602827	0.586696	0.549822	0	0		0.48618		
Isoproterenol 500uM	500000	0.505705	0.505436	0.600133	0.611908	0.531388	0.428744	0.455444	0.56219	0.452828	0	0		0.423071		
Isoproterenol 50uM	50000	0.69008	0.427835	0.363097	0.506611	0.287074	0.30101	0.238809	0.4337	0.361595	0.22787	0.399463	0.520767	0.396493	0.132411	0.276989
Isoproterenol 5uM	5000	0.573857	0.357813	0.289166	0.421053	0.13344	0.240197	0.138893	0.274154	0.296574	0.18943	0.355666	0.481964	0.312684	0.134018	0.266197
Isoproterenol 500nM	500	0.3233	0.146472	0.233869	0.241755	0.083287	0.173743	0.088176	0.167332	0.214393	0.147499	0.268123	0.336092	0.202004	0.082528	0.34314
Isoproterenol 50nM	50	-0.02209	0.020922	0.069656	0.036547	0.031046	0.013052	-0.00972	0.021185	0.025565	0	0		0.016924		
Isoproterenol 5nM	5	-0.02169	0.001455	0.015022	-0.00883	0.011616	-0.02416	-0.01661	-0.03351	0.001119	0	0		-0.00687		
Adrenaline 1mM	1000000	0.438058	0.588412	0.654646	0.651939	0.621838	0.545607	0.56437	0.541464	0.531454	0	0		0.467072		
Adrenaline 100uM	100000	0.577236	0.440056	0.519117	0.607661	0.446216	0.341682	0.390337	0.602928	0.363253	0.48952	0.535941	0.63866	0.496051	0.100559	0.194535
Adrenaline 10uM	10000	0.520062	0.337168	0.263434	0.381105	0.163302	0.205381	0.147584	0.273239	0.299688	0.203486	0.31466	0.446966	0.296339	0.112927	0.196221
Adrenaline 1uM	1000	0.240591	0.118109	0.134269	0.188301	0.076393	0.111531	0.061781	0.114963	0.135967	0.109947	0.108816	0.183192	0.131988	0.050047	0.31423
Adrenaline 100nM	100	-0.00672	0.023303	0.009842	0.030431	0.040758	-0.00826	0.005414	0.006605	0.021948	0	0		0.011212		
Adrenaline 10nM	10	-0.00639	0.010954	0.003866	0.021449	0.040138	-0.01987	-0.00834	-0.00039	0.020845	0	0		0.005661		
Adrenaline 1nM	1	-0.04256	0.002028	0.01005	0.008151	0.031315	-0.00721	-0.02067	-0.01	-0.00087	0	0		-0.00271		

20 min

		1	2	3	4	5	6		
		S012-4	S013-4	S014-4	S023-4	S039-4	S040-4	Ave.	SD
Isoproterenol 5mM	5000000	0.623385	0.654316	0.584601	0.610575			0.618219	0.028973
Isoproterenol 500uM	500000	0.646508	0.624868	0.472714	0.521665			0.566439	0.082894
Isoproterenol 50uM	50000	0.49949	0.512581	0.320096	0.63444	0.161777	0.361885	0.491652	0.167788
Isoproterenol 5uM	5000	0.427416	0.409776	0.29738	0.498414	0.099219	0.337615	0.408247	0.139467
Isoproterenol 500nM	500	0.237883	0.257341	0.070348	0.2762	0.096651	0.335175	0.210443	0.105252
Isoproterenol 50nM	50	-0.009	-0.01359	-0.01459	-0.00668			-0.01096	0.003753
Isoproterenol 5nM	5	-0.05393	-0.08247	-0.06695	-0.03238			-0.05893	0.021201
Adrenaline 1mM	1000000	0.63069	0.687409	0.578632	0.586492			0.620806	0.049966
Adrenaline 100uM	100000	0.634549	0.611666	0.409991	0.678708	0.471462	0.598386	0.583728	0.10373
Adrenaline 10uM	10000	0.410303	0.441084	0.274646	0.51023	0.135033	0.391139	0.409066	0.134564
Adrenaline 1uM	1000	0.209038	0.221573	0.155841	0.222399	-0.00281	0.181658	0.202213	0.085972
Adrenaline 100nM	100	0.006968	0.041265	-0.0078	0.032168			0.018151	0.022576
Adrenaline 10nM	10	-0.00092	0.001971	-0.00281	0.018767			0.004251	0.009875
Adrenaline 1nM	1	-0.01392	-0.01575	-0.01273	0.01574			-0.00667	0.014989

20 min		C36-4	C37-4	C38-4	C040-4	C41-4	C43-4	C46-4	C47-4	C048-4	C050-4	C051-4	C053-4	Average	SD	T-test
Isoproterenol 5mM	5000000	0.48141	0.59351	0.6557	0.642971	0.629867	0.568406	0.59554	0.552801	0.563449	0	0	0	0.440304		
Isoproterenol 500uM	500000	0.487475	0.517069	0.651278	0.57275	0.533343	0.423929	0.444228	0.527197	0.443848	0	0	0	0.383426		
Isoproterenol 50uM	50000	0.629321	0.612423	0.449734	0.403493	0.298672	0.319218	0.26333	0.4659	0.421751	0.279892	0.469228	0.464759	0.423144	0.119647	0.459322
Isoproterenol 5uM	5000	0.550668	0.548669	0.333733	0.322789	0.140863	0.281765	0.157021	0.308233	0.314989	0.2479	0.392549	0.375606	0.331232	0.127212	0.421843
Isoproterenol 500nM	500	0.259487	0.222269	0.310466	0.177412	0.069729	0.192632	0.066295	0.220416	0.214187	0.17775	0.273197	0.278378	0.205185	0.076242	0.443569
Isoproterenol 50nM	50	-0.01954	-0.02067	0.031378	0.047046	-0.00315	0.004184	-0.06001	0.017568	0.002853	0	0	0	-2.8E-05		
Isoproterenol 5nM	5	-0.02733	-0.04237	-0.06312	-0.01285	0.005228	-0.03773	-0.05439	-0.09231	-0.05387	0	0	0	-0.03156		
Adrenaline 1mM	1000000	0.463035	0.57392	0.680409	0.654641	0.64785	0.5609	0.575303	0.54353	0.533229	0	0	0	0.436068		
Adrenaline 100uM	100000	0.576932	0.51415	0.633898	0.547847	0.467596	0.370675	0.416147	0.619397	0.379071	0.49629	0.572353	0.5781	0.514371	0.089895	0.156635
Adrenaline 10uM	10000	0.571906	0.562986	0.336593	0.277317	0.201285	0.276292	0.171319	0.309766	0.304976	0.222378	0.402894	0.370702	0.334034	0.127794	0.349466
Adrenaline 1uM	1000	0.236336	0.199332	0.147867	0.138014	0.11307	0.143043	0.071892	0.159119	0.144688	0.123798	0.129612	0.128095	0.144572	0.041439	0.303851
Adrenaline 100nM	100	0.032552	-0.00636	0.019104	0.015082	0.053104	0.0114	-0.01779	0.027575	0.018305	0	0	0	0.012748		
Adrenaline 10nM	10	-0.01803	0.00832	0.009432	0.025275	0.052211	-0.00486	-0.01361	0.039907	0.006963	0	0	0	0.0088		
Adrenaline 1nM	1	-0.0102	-0.01384	0.014224	0.004534	0.037719	-0.00633	-0.03471	0.040481	-0.02203	0	0	0	0.000821		
