# Supplementary material for: Neurally mediated syncope diagnosis based on adenylate cyclase activity in Japanese patients
Source: PLoS One. 2019 Apr 18;14(4):e0214733. doi: 10.1371/journal.pone.0214733 (PMC6472876; doi:10.1371/journal.pone.0214733)
Supplement: S7 Table — Upper: Adrenaline Lower: Isoproterenol. (PDF) [file pone.0214733.s007.pdf]

S7 Table. The raw data of adenylate cyclase activities in the different seasons from three volunteers by adrenaline (AD) and isoproterenol (IP).

		(A) C38	(A) C38	(B) C11	(B) C11	(C) C41	(C) C41
		20150817	20160707	20150605	20151109	20160715	20160929
Adrenaline 1mM	1mM	0.67057	0.680594	0.665778	0.606962	0.681196	0.653925
Adrenaline 100uM	100uM	0.540641	0.506677	0.382489	0.437801	0.439848	0.458885
Adrenaline 10uM	10uM	0.23604	0.261932	0.180688	0.173245	0.090222	0.111025
Adrenaline 1uM	1uM	0.13971	0.113756	0.107534	0.108717	0.017669	0.07354
Adrenaline 100nM	100nM	0.039614	-0.00944	0.00513	0.01664	-0.00522	0.044332
Adrenaline 10nM	10nM	0.015129	0.035078	0.000772	-0.03691	-0.0155	0.020914
Adrenaline 1nM	1nM	0.046947	0.011143	0.019697	-0.02545	0.000881	0.008347

		(A) C38	(A) C38	(B) C11	(B) C11	(C) C41	(C) C41
		20150817	20160707	20150605	20151109	20160715	20160929
Isopreterenol 5mM	5mM	0.67403	0.695884	0.669095	0.653095	0.704314	0.678168
Isopreterenol 500uM	500uM	0.650422	0.627609	0.608009	0.585391	0.588315	0.549981
Isopreterenol 50uM	50uM	0.414308	0.351528	0.288006	0.340537	0.20909	0.216537
Isopreterenol 5uM	5uM	0.24785	0.255737	0.188182	0.193463	0.079477	0.113567
Isopreterenol 500nM	500nM	0.192454	0.193664	0.123776	0.123229	0.013094	0.10231
Isopreterenol 50nM	50nM	0.054565	0.05684	0.037773	-0.00865	-0.03929	0.030603
Isopreterenol 5nM	5nM	0.026084	-0.04086	0.010342	-0.02485	-0.04243	0.021786
