# Supplementary material for: Neurally mediated syncope diagnosis based on adenylate cyclase activity in Japanese patients
Source: PLoS One. 2019 Apr 18;14(4):e0214733. doi: 10.1371/journal.pone.0214733 (PMC6472876; doi:10.1371/journal.pone.0214733)
Supplement: S8 Table — The AC activities in the different seasons from the healthy volunteer during the HUT test. HUT was tested in two different seasons on the 15th of July and the 29th of September. Upper: Adrenaline Lower: Isoproterenol. (PDF) [file pone.0214733.s008.pdf]

S8 Table. The raw data of adenylate cyclase activities in the different seasons from C41 volunteer by adrenaline (AD) and isoproterenol (IP).

Baseline	2016/7/15_C 41-1	2016/9/29_C41-1
Adrenaline 1 mM	0.681196113	0.653925033
Adrenaline 100 uM	0.439847737	0.458885064
Adrenaline 10 uM	0.090222385	0.11102539
Adrenaline 1 uM	0.017669163	0.073539919
Adrenaline 100 nM	-0.005222152	0.044331786
Adrenaline 10 nM	-0.015496291	0.020913697
Adrenaline 1 nM	0.000881167	0.008346955
	2016/7/15_C 41-1	2016/9/29_C41-1
Isoproterenol 5 mM	0.70431352	0.678167639
Isoproterenol 500 uM	0.588314875	0.549981374
Isoproterenol 50 uM	0.20909015	0.216537372
Isoproterenol 5 uM	0.079477021	0.11356683
Isoproterenol 500 nM	0.013093756	0.102309993
Isoproterenol 50 nM	-0.039294357	0.030603126
Isoproterenol 5 nM	-0.042426339	0.021785949

70°	2016/7/15_C 41-2	2016/9/29_C41-2
Adrenaline 1 mM	0.577939833	0.631303649
Adrenaline 100 uM	0.410099775	0.412418639
Adrenaline 10 uM	0.131509913	0.104945267
Adrenaline 1 uM	0.06308888	0.072935686
Adrenaline 100 nM	-0.006361456	0.028093621
Adrenaline 10 nM	-0.006748363	0.026008587
Adrenaline 1 nM	-0.030284004	0.009360127
	2016/7/15_C 41-2	2016/9/29_C41-2
Isoproterenol 5 mM	0.604583599	0.657979519
Isoproterenol 500 uM	0.525597604	0.519892938
Isoproterenol 50 uM	0.227263518	0.164946313
Isoproterenol 5 uM	0.131486938	0.111924559
Isoproterenol 500 nM	0.09318862	0.054601732
Isoproterenol 50 nM	-0.014381743	0.02607083
Isoproterenol 5 nM	-0.021363537	-0.004393549

after 10 minutes	2016/7/15_C 41-3	2016/9/29_C41-3
Adrenaline 1 mM	0.5692913	0.621837861
Adrenaline 100 uM	0.442314865	0.446216021
Adrenaline 10 uM	0.133840085	0.163301567
Adrenaline 1 uM	0.062601684	0.076393392
Adrenaline 100 nM	0.007657054	0.040757942
Adrenaline 10 nM	0.001580496	0.040138371
Adrenaline 1 nM	-0.006248661	0.031314539
	2016/7/15_C 41-3	2016/9/29_C41-3
Isoproterenol 5 mM	0.588528336	0.651187329
Isoproterenol 500 uM	0.53169037	0.531388019
Isoproterenol 50 uM	0.30250403	0.287074222
Isoproterenol 5 uM	0.156699226	0.133440262
Isoproterenol 500 nM	0.081058452	0.08328681
Isoproterenol 50 nM	0.018682844	0.031046119
Isoproterenol 5 nM	-0.01026813	0.011616039

after 20 minutes	2016/7/15_C 41-4	2016/9/29_C41-4
Adrenaline 1 mM	0.571094134	0.647850346
Adrenaline 100 uM	0.435057911	0.467595924
Adrenaline 10 uM	0.197732676	0.201285095
Adrenaline 1 uM	0.088809572	0.113070168
Adrenaline 100 nM	0.045535149	0.05310383
Adrenaline 10 nM	0.032084405	0.052211269
Adrenaline 1 nM	0.02304824	0.037719033
	2016/7/15_C 41-4	2016/9/29_C41-4
Isoproterenol 5 mM	0.604432722	0.629867318
Isoproterenol 500 uM	0.512612186	0.533342781
Isoproterenol 50 uM	0.343663139	0.298672273
Isoproterenol 5 uM	0.204952943	0.140863379
Isoproterenol 500 nM	0.117974675	0.069728912
Isoproterenol 50 nM	0.041354539	-0.00315075
Isoproterenol 5 nM	0.013480592	0.005228001
